# Can integration of Alzheimer’s plasma biomarkers with MRI, cardiovascular, genetics, and lifestyle measures improve cognition prediction?

**DOI:** 10.1093/braincomms/fcae300

**Published:** 2024-09-04

**Authors:** Robel K Gebre, Jonathan Graff-Radford, Vijay K Ramanan, Sheelakumari Raghavan, Ekaterina I Hofrenning, Scott A Przybelski, Aivi T Nguyen, Timothy G Lesnick, Jeffrey L Gunter, Alicia Algeciras-Schimnich, David S Knopman, Mary M Machulda, Maria Vassilaki, Val J Lowe, Clifford R Jack, Ronald C Petersen, Prashanthi Vemuri

**Affiliations:** Department of Radiology, Mayo Clinic, Rochester, MN 55905, USA; Department of Neurology, Mayo Clinic, Rochester, MN 55905, USA; Department of Neurology, Mayo Clinic, Rochester, MN 55905, USA; Department of Radiology, Mayo Clinic, Rochester, MN 55905, USA; Department of Quantitative Health Sciences, Mayo Clinic, Rochester, MN 55905, USA; Department of Quantitative Health Sciences, Mayo Clinic, Rochester, MN 55905, USA; Department of Laboratory Medicine and Pathology, Mayo Clinic, Rochester, MN 55905, USA; Department of Quantitative Health Sciences, Mayo Clinic, Rochester, MN 55905, USA; Department of Radiology, Mayo Clinic, Rochester, MN 55905, USA; Department of Laboratory Medicine, Mayo Clinic, Rochester, MN 55905, USA; Department of Neurology, Mayo Clinic, Rochester, MN 55905, USA; Department of Psychology, Mayo Clinic, Rochester, MN 55905, USA; Department of Quantitative Health Sciences, Mayo Clinic, Rochester, MN 55905, USA; Department of Radiology, Mayo Clinic, Rochester, MN 55905, USA; Department of Radiology, Mayo Clinic, Rochester, MN 55905, USA; Department of Neurology, Mayo Clinic, Rochester, MN 55905, USA; Department of Radiology, Mayo Clinic, Rochester, MN 55905, USA

**Keywords:** Alzheimer’s disease, plasma biomarkers, cognition, machine learning, aging

## Abstract

There is increasing interest in Alzheimer’s disease related plasma biomarkers due to their accessibility and scalability. We hypothesized that integrating plasma biomarkers with other commonly used and available participant data (MRI, cardiovascular factors, lifestyle, genetics) using machine learning (ML) models can improve individual prediction of cognitive outcomes. Further, our goal was to evaluate the heterogeneity of these predictors across different age strata. This longitudinal study included 1185 participants from the Mayo Clinic Study of Aging who had complete plasma analyte work-up at baseline. We used the Quanterix Simoa immunoassay to measure neurofilament light, Aβ_1–42_ and Aβ_1–40_ (used as Aβ_42_/Aβ_40_ ratio), glial fibrillary acidic protein, and phosphorylated tau 181 (p-tau181). Participants’ brain health was evaluated through gray and white matter structural MRIs. The study also considered cardiovascular factors (hyperlipidemia, hypertension, stroke, diabetes, chronic kidney disease), lifestyle factors (area deprivation index, body mass index, cognitive and physical activities), and genetic factors (*APOE*, single nucleotide polymorphisms, and polygenic risk scores). An ML model was developed to predict cognitive outcomes at baseline and decline (slope). Three models were created: a base model with groups of risk factors as predictors, an enhanced model included socio-demographics, and a final enhanced model by incorporating plasma and socio-demographics into the base models. Models were explained for three age strata: younger than 65 years, 65–80 years, and older than 80 years, and further divided based on amyloid positivity status. Regardless of amyloid status the plasma biomarkers showed comparable performance (*R*² = 0.15) to MRI (*R*² = 0.18) and cardiovascular measures (*R*² = 0.10) when predicting cognitive decline. Inclusion of cardiovascular or MRI measures with plasma in the presence of socio-demographic improved cognitive decline prediction (*R*² = 0.26 and 0.27). For amyloid positive individuals Aβ_42_/Aβ_40_, glial fibrillary acidic protein and p-tau181 were the top predictors of cognitive decline while Aβ_42_/Aβ_40_ was prominent for amyloid negative participants across all age groups. Socio-demographics explained a large portion of the variance in the amyloid negative individuals while the plasma biomarkers predominantly explained the variance in amyloid positive individuals (21% to 37% from the younger to the older age group). Plasma biomarkers performed similarly to MRI and cardiovascular measures when predicting cognitive outcomes and combining them with either measure resulted in better performance. Top predictors were heterogeneous between cross-sectional and longitudinal cognition models, across age groups, and amyloid status. Multimodal approaches will enhance the usefulness of plasma biomarkers through careful considerations of a study population’s socio-demographics, brain and cardiovascular health.

## Teaser

Gebre *et al*. report combining plasma biomarkers with brain or cardiovascular health measures can improve cognitive decline prediction. Glial fibrillary acidic protein and phosphorylated tau 181 were associated with cognitive decline in amyloid positive individuals. Multimodal approaches will enhance usefulness of plasma biomarkers, considering population socio-demographics, brain and cardiovascular health.

## Graphical Abstract

**Graphical abstract:**
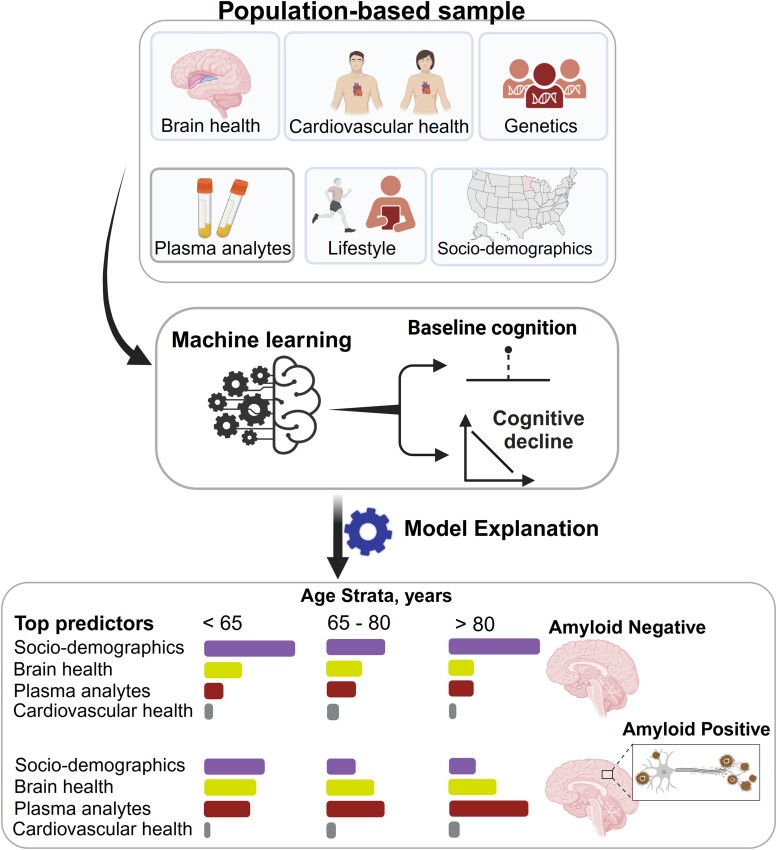

## Introduction

Prediction of longitudinal cognitive decline and identification of its risk factors is crucial for timely interventions and for formulating individualized treatment strategies. In late-onset Alzheimer’s disease (AD), cognition can be influenced by several factors such as morphology of the gray matter cortex,^1,2^ white matter integrity^3^ and hippocampal volume.^4^ Certain genes such as apolipoprotein E (*APOE*) have been shown to influence memory and cognition.^5-7^ Education and lifestyle factors such as area deprivation index (ADI) are also associated with cognitive function.^8,9^ Recently, AD plasma biomarkers have emerged as potential predictors of cross-sectional cognitive outcomes and long-term cognitive decline. The application of plasma markers for dementia diagnosis and clinical trial design promises a paradigm shift due its cost-effectiveness and low burden on participants compared with MRI and PET imaging. However, considering the heterogeneous nature of cognition, addressing the efficacy of plasma biomarkers compared with the presence of other well-known risk factors has not been investigated.

The main AD-related plasma biomarkers are Aβ_42_ or Aβ_40_ and the phosphorylated tau marker (p-tau) at threonine 181, 217, 231.^10-12^ There are also dementia-non-specific plasma analytes that are related to neurodegeneration and neuroinflammation such as the neurofilament light (NfL) associated with axonal integrity,^13^ and glial fibrillary acidic protein (GFAP) which is an indicator of astroglial activation and astrocytosis. Prior studies have also shown that Aβ_42_/Aβ_40_ can predict amyloid burden^12^ and plasma p-tau can predict neuropathology in AD.^14,15^ In addition to its association to risk of AD^16^ and AD pathology,^17^ GFAP had been shown to associate with vascular pathology.^18,19^ A combination of one or more of these AD-related plasma biomarkers has been shown to predict cognitive decline.^20-22^ Nonetheless, unaddressed questions remain, such as, how does the variance explained by plasma biomarkers compare with the variance captured by imaging and non-imaging measures when predicting cognitive decline? Are plasma biomarkers equally predictive of cognitive decline for different age groups compared with the imaging and non-imaging predictors of cognitive decline? Hence, this study posits two objectives: (i) elucidate the utility of plasma biomarkers in comparison to brain health, cardiovascular, genetics and lifestyle measures, and (ii) understand the heterogeneity that drives cognitive decline for different age groups with a comparison of their performance to measures of brain health, cardiovascular, genetics and lifestyle.

## Materials and methods

### Study participants

Participants enrolled in Mayo Clinic Study of Aging (MCSA) were included in the study. The MCSA is a longitudinal population-based study of cognitive aging among residents of Olmsted County, Minnesota, USA.^23^

The MCSA started in 2004 with every participant requiring a clinical visit at baseline which had cognitive, lifestyle and genetics information available along with stored plasma samples and followed up through a clinical visit every 15 months. A subset of participants agreed to take part in MRI and PET studies which was conducted very close in time to the clinical visit. When AD plasma biomarkers were introduced, plasma analyte measurements were performed on the samples at least once for all participants which may or may not correspond to imaging visits. For this study, we selected all participants who had the first available processed plasma measurements. The inclusion criteria were availability of complete plasma analyte work-up, cognition assessment, amyloid PET scan, and a clinical diagnosis of cognitively unimpaired.

### Cognition measure

We used the global cognition *z*-score as the outcome measure for the machine learning (ML) models. This score is comprised of the executive, language, memory and visuospatial cognitive domains.^24,25^ These four domains were evaluated using nine tests; Trail Making Test Part B and Wechsler Adult Intelligence Scale–Revised Digit Symbol for executive, Boston Naming Test and category fluency for language, Wechsler Memory Scale–Revised Logical Memory II for delayed recall, Wechsler Memory Scale–Revised Visual Reproduction II also for delayed recall, and Auditory Learning Verbal Test delayed recall for memory and Wechsler Adult Intelligence Scale–Revised Picture Completion and Block Design for visuospatial performance.^24,25^

### Plasma analytes

The plasma biomarkers included in the study were GFAP, phosphorylated tau (p-tau181), NfL, Aβ_1–42_, and Aβ_1–40_. Participants had their blood drawn following an overnight fast. Samples were processed by centrifugation, divided into aliquots, and stored at −80°C. The plasma p-tau181 was measured on stored samples using the Quanterix Simoa Advantage V2 kit (item #103714). This assay uses a mouse monoclonal antibody (AT270) specific for the threonine-181 phosphorylation site of human tau protein. Plasma Aβ_1–42_, Aβ_1–40_, GFAP and NfL were measured using the Quanterix Neurology 4-Plex E Advantage kit (N4PE, item #103670). Both kits were used according to manufacturer’s instructions and ran on a Quanterix HD-X analyzer (Quanterix Corporation). Details on sample preparate on can be found in V. K Ramanan *et al*.,^26^ Mielke *et al*.^27^ and Jack *et al*.^28^

### Imaging methods

#### MRI (T1, FLAIR, diffusion)

The T1-weighted and FLAIR MRI were acquired using standard structural magnetization-prepared rapid acquisition gradient echo (MPRAGE) and FLAIR sequences.^29^ We included cortical thicknesses (CTh), hippocampal volume (HVA) adjusted for TIV, ventricular volume, volume of the corpus callosum (CC) and total intracranial volume (TIV) from T1 images. The CTh were grouped into five regions of interest temporal, cingulate, parietal, frontal and occipital.^30^ We used white matter hyperintensity as a fraction of TIV (WMH) from FLAIR. We used fractional anisotropy (FA) of white matter tracts of the body of CC (BCC), cingulate gyrus part of cingulum (CGC), cingulum hippocampus (CGH) and genu of CC (GCC) as indicators of white matter integrity and health. A detailed description of the acquisition sequence and preprocessing for the WMH and FA has been published previously.^31,32^

#### Amyloid PET

Participants were scanned using the ^11^C-Pittsburgh compound B (PiB) and the standard uptake value ratio (SUVR) was normalized to the cerebellar crus gray matter.^33^ The global cortical amyloid median uptake in the prefrontal, orbitofrontal, parietal, temporal, anterior cingulate and posterior cingulate/precuneus regions were included to calculate the SUVRs. The cut-point for amyloid PET positivity was chosen at the global cortical SUVR ≥ 1.52 (25 Centiloids) to capture most of the amyloid positive scans.^34-36^

#### Cardiovascular measures

Cardiovascular health measures were assessed from the electronic health care records and the MCSA study visit. Chronic kidney disease (CKD), congestive heart failure (CHF), coronary artery disease (CAD), hypertension, hyperlipidemia, diabetes, stroke, cardiac arrythmias variables were recorded by nurse abstraction from the electronic health care records and systolic and diastolic blood pressures are recorded at each MCSA clinic visit.

#### Lifestyle measures

Lifestyle was measured using physical and cognitive activities (PhyAct and CogAct) from self-reported questionnaires,^37^ national ranking of the ADI which is a measure of the area-level socio-economic deprivation, and body mass index (BMI). In this study we included ADI with demographics, and together referred as socio-demographics.

#### Genetics measures

Genetics was represented with the single nucleotide polymorphisms (SNPs) tagging *APOE*-ԑ4 (rs429358) and *APOE*-ԑ2 (rs7412), as well as polygenic risk scores (PRSs) calculated from SNPs associated with clinically diagnosed AD dementia.^26^

### ML experiments

We conducted two major experiments: (i) prediction of baseline cognition and (ii) prediction of long-term cognitive decline. The target labels for the baseline models were the baseline global cognition *z*-scores. For the long-term prediction models, we calculated individual annualized slopes from baseline and used these slopes for cognitive decline models. The input predictors in both experiments were baseline measurements only.

#### Modeling strategies

We categorized the predictors into six components: socio-demographics, plasma, MRI, cardiovascular, genetics and lifestyle. We trained a model for each component separately.

To better understand the performance of the six categories, we first modeled each component without socio-demographics to serve as our base models, and then we added socio-demographics. Although it could be argued that socio-demographics should serve as the base model, we separately trained base models per biomarker category since our aim was explorative seeking to elucidating the utility of plasma biomarkers compared with the others when predicting cross-sectional and longitudinal cognitive outcomes. Furthermore, we combined these base category models with plasma and socio-demographic measures into advanced models for further exploration. Each model was then repeated based on amyloid positivity status which was used only as a modeling criterion. Amyloid PiB SUVR values were not used as inputs in the modeling. The rationale behind this approach was to compare the performance of plasma biomarkers when participants where on the AD pathway versus not. This separation and repeating of the analysis shed light on both the mechanisms underlying cognitive outcomes and allowed comparison of predictors important for participants on the AD pathway versus non-AD pathway. Furthermore, conducting separate models between amyloid positive and negative participants was necessary to understand the diagnostic value of plasma biomarkers to predict cognition based on amyloid status.

The model interpretation was performed based on three age groups: 65 years old and younger, between 65 and 80 years old, and older than 80 years old. The age grouping was chosen to reflect a younger, middle age, and oldest old in the population.

#### Preprocessing

A repeated randomized stratified *k*-fold cross-validation (*k* = 5-folds, n=10repeats; k×n=50totalsplits) training scheme was adapted. In each of the 50 random splits, the data was divided into 80% training set and 20% testing set. Missing data was handled by conducting an iterative imputation using a Bayesian Ridge regressor^38^ independently on the training and testing splits to avoid data leakage. The training data was largely intact except for a few categories: the white matter features (<12%), followed by the lifestyle category (<9%), then the genetics features (<5%), and all other categories had less than 1% or no missing data (Supplementary Fig. 1). Lastly, to obtain a normal distribution for plasma p-tau181, GFAP, NfL and MRI WHM, the natural log transformation was applied^26^ (Supplementary Fig. 2).

#### Model training

We used Autogluon package (*version 1.0.0*) which is an automatic machine learning (AutoML) framework.^39^ Briefly, this framework uses a multi-layer stack ensemble of different ML models with repeated *k*-fold bagging. We trained a variety of models such as gradient boosted models (GBM), XGboost, CatBoost, Random Forest, Extra Trees and K-nearest neighbors, followed by a weighted ensemble. By combining our preprocessing steps with Autogluon’s framework, we aimed to randomize the data as much as possible while minimizing overfitting which is a common problem when modeling with real world data. Then for model interpretation the best model was selected based on the highest coefficient of determination (*R*²)^40^ on the testing set while maintaining the smallest performance gap between the training and testing sets.

#### Explaining model predictions

After training, to explain the best model, we implemented both permutation feature importance and SHAP (SHapley Additive exPlanations).^41^ Permutation feature importance works by calculating the decrease in a model’s performance when an input is taken out as a predictor.^42^ Due to drawbacks of permutation feature importance especially with multicollinearity, we primarily used SHAP and referred to permutation importance only for crosscheck validation purposes.

SHAP is based on cooperative game theory and works by quantifying the contribution of an input for a model prediction. There are many types of explainable AI (XAI) methods, however, we chose SHAP due to its better performance as shown by previous works compared with others such as breakDown^43^ and locally interpretable model agnostic explanations (LIME).^44^ A recent systematic review of known XAI methods has also shown that SHAP maintains a greater balance between stability and reliability compared with other methods.^45^ Lastly, pie charts were used to visualize age-stratified feature contributions. In these charts, the size of each wedge represents the median importance for each group calculated from the SHAP values of the features within that age-strata normalized by the group size to allow for groupwise comparisons.

### Statistical analyses

Comparison of means between the amyloid positive and negative groups was conducted at different stages of the study. Normality was checked using Chapiro–Wilk test before running a mean comparison using either two-sided *t*-test for parametric distribution or a Mann–Whitney *U*-test for non-parametric. When comparing categorical variables, the Chi-squared test was used. In addition, the effect size for the mean comparisons was determined using Cohen’s *d*.

## Results

There were 1185 participants at baseline (age mean [range]: 67.63 years [31.77–86.18]). Exactly five follow-ups (*n* = 682) from baseline for a total of six visits were selected in the longitudinal cognitive decline (slope) models. The average follow-up time for the six visits was 6.67 years [SD: 0.73 years] (Table 1).

**Table 1:** Descriptive statistics (*n*, mean [SD] or %) of study participants and the plasma biomarkers used in the study

	All	Aβ+	Aβ−	*P*-value
Number of participants, *n*	1185	225	960	
**Age groups, *n***				
≤65 years	460	20	440	
65–80 years	563	125	418	
>80 years	182	80	102	
Follow-up time (6 visits), mean [SD] years	6.67 [0.73]	6.73 [0.83]	6.66 [0.71]	0.30
Cognition *z*-scores, mean [SD]	0.37 [0.92]	−0.04 [0.90]	0.47 [0.90]	<0.001*
**Cognition *z*-scores in age groups, mean [SD]**				
≤65 years	0.87 [0.77]	0.66 [0.82]	0.88 [0.77]	0.12
65–80 years	0.19 [0.82]	0.099 [0.83]	0.22 [0.81]	0.15
>80 years	−0.35 [0.90]	−0.41 [0.89]	−0.30 [0.91]	0.37
**Socio-demographics**				
Sex, male %	53%	52%	53%	0.91
Age, years, mean [SD]	67.63 [12.14]	75.41 [7.30]	65.37 [12.26]	<0.001*
Education, mean [SD]	14.99 [2.54]	14.73 [2.72]	15.04 [2.49]	0.13
ADI, mean [SD]	40.56 [17.00]	42.67 [16.78]	40.06 [17.03]	0.04
**Plasma, mean [SD] [pg/mL]**				
lnGFAP ^a^	4.45 [0.55]	4.82 [0.46]	4.34 [0.52]	<0.01*
lnNfL	2.97 [0.61]	3.27 [0.54]	2.88 [0.59]	<0.01*
$A\beta_{42}/A\beta_{40}$ (x100)	6.03 [1.31]	5.29 [1.11]	6.20 [1.29]	<0.001*
lnp-tau181	0.54 [0.50]	0.87 [0.45]	0.46 [0.48]	<0.001*

Participants are categorized into amyloid positive (Aβ+) and amyloid negative (Aβ−) based on amyloid PET PIB SUVR cut-point of 1.52 (25 Centiloids). All participants were clinically cognitively unimpaired at baseline. The comparisons shown between the Aβ+ and Aβ− participants are mean comparisons using either Mann Whitney U-test or two-tailed independent *t*-test depending on normality if the variable is continuous or Chi-squared test if the variable is categorical. Statistical significance was set at a *P* < 0.05.

^*^ *P*-value < 0.05.

^a^ln: natural logarithm of the raw values applied for normalizing the distribution of skewed variables.

**Figure 1:**
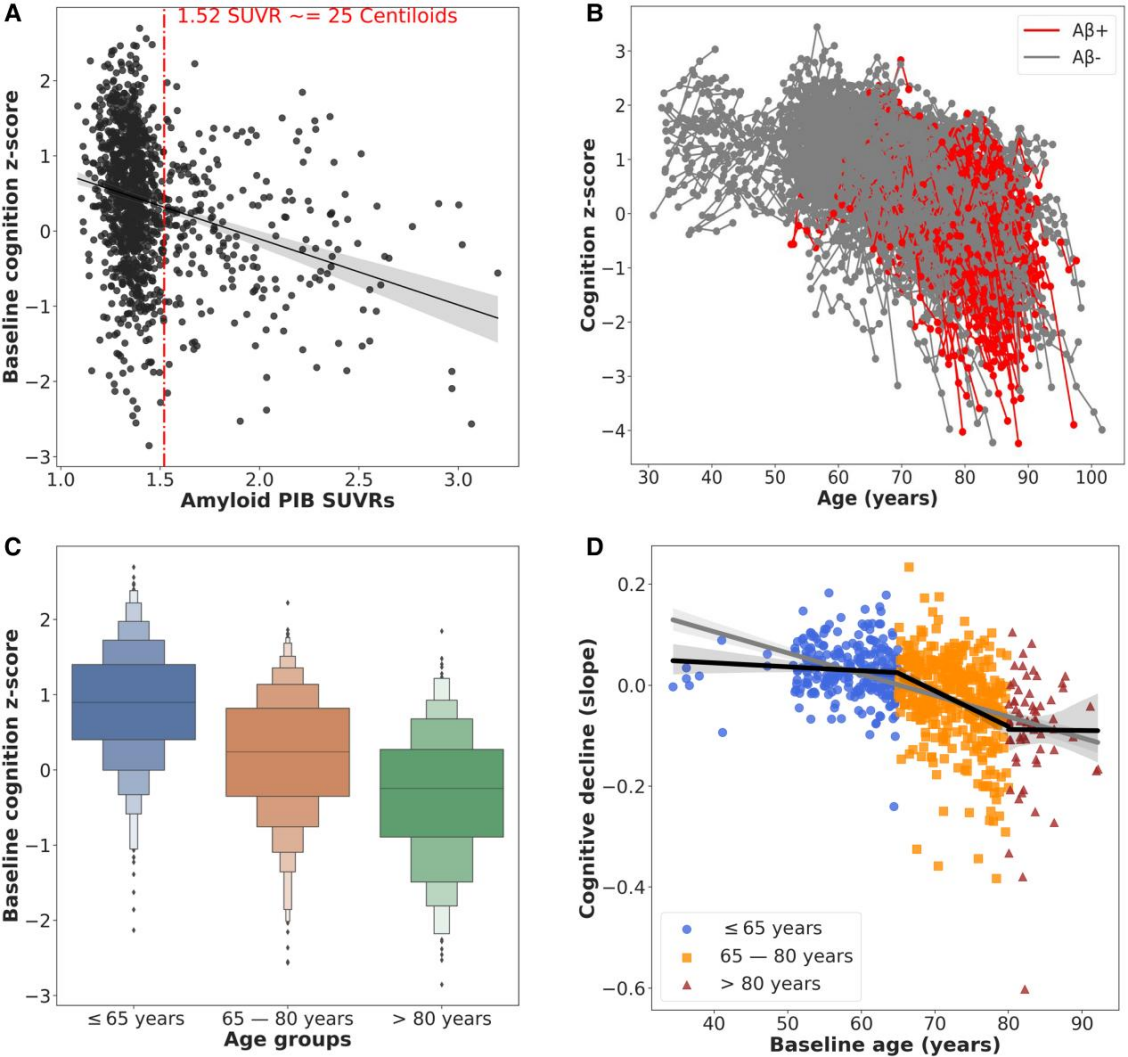
**Characteristics of cognition outcomes by amyloid status (A and B), and age strata (B and C).** In (**A**) the cut-point of the amyloid PIB SUVR values (dashed line) against the baseline cognition *z*-scores are shown with a regression fit to show the characteristic trend, (**B**) shows the cognition *z*-scores for all participants longitudinally across age highlighted by amyloid status, (**C**) shows boxplots of the baseline cognition *z*-scores across the age groups, and (**D**) shows the cognitive decline (slopes) for the different age groups fitted with regression lines within each group and overall to show the trend of the decline. Aβ, beta amyloid.

Cognition declined with increasing amyloid PIB SUVRs across the cohort. Amyloid positive (Aβ+) participants showed a faster decrease in cognition *z*-scores compared with negatives (Aβ−), and younger participants had better baseline cognition *z*-scores and reduced cognitive decline (slopes) compared with older participants (Fig. 1).

All the plasma analytes were significantly different between Aβ+ and Aβ− participants (*P* < 0.05) (Table 1). Amongst the brain health measures only TIV and FA SCC did not show significant mean differences between Aβ+ and Aβ− participants (*P* > 0.05). On the contrary, for the cardiovascular health measures, only Diastolic and Systolic BP showed significant mean differences between Aβ+ and Aβ− participants (*P* < 0.05). The genetics measures also did not show significant differences based on amyloid status. Lastly, for the lifestyle measures only BMI showed significant mean differences (*P* < 0.05), with cognitive activity showing borderline significant difference (*P* = 0.05) between Aβ+ and Aβ− participants (Table 2).

**Table 2:** Description of other predictors used in the study

	All	Aβ+	Aβ−	*P*-value
**Brain health measures, mean [SD]**				
CTh Cingulate [mm]	2.49 [0.14]	2.47 [0.14]	2.50 [0.14]	<0.01*
CTh Parietal [mm]	2.12 [0.13]	2.06 [0.12]	2.13 [0.13]	<0.01*
CTh Frontal [mm]	2.38 [0.13]	2.32 [0.12]	2.39 [0.13]	<0.01*
CTh Occipital [mm]	1.82 [0.11]	1.78 [0.10]	1.83 [0.11]	<0.01*
Temporal Meta-ROI [mm]	2.90 [0.16]	2.83 [0.16]	2.92 [0.15]	<0.01*
lnWMH ^a^ [TIV adjusted]	−5.37 [0.92]	0.01 [0.01]	0.01 [0.01]	<0.01*
HVA [TIV adjusted]	−0.25 [0.60]	−0.42 [0.62]	−0.20 [0.58]	<0.01*
TIV [cm^3^×10^3^]	1.50 [0.16]	1.51 [0.15]	1.50 [0.15]	**0**.**85**
Ventricular volume [cm^3^]	38.93 [19.36]	46.54 [20.85]	36.72 [18.34]	<0.01*
CC volume [cm^3^]	0.55 [0.10]	0.53 [0.09]	0.55 [0.09]	<0.01*
FA GCC	0.61 [0.05]	0.59 [0.05]	0.61 [0.04]	<0.01*
FA BCC	0.60 [0.04]	0.59 [0.05]	0.60 [0.04]	<0.01*
FA SCC	0.69 [0.04]	0.69 [0.04]	0.69 [0.03]	**0**.**62**
FA CGC	0.50 [0.04]	0.49 [0.03]	0.50 [0.03]	0.04*
FA CGH	0.47 [0.03]	0.46 [0.03]	0.47 [0.03]	<0.01*
**Cardiovascular health measures**				
CKD, healthy %	94.18	89.51	95.53	0.66
CHF, healthy %	96.12	94.01	96.73	1.0
CAD, healthy %	78.73	65.54	82.57	0.13
Hypertension, healthy %	44.81	28.46	49.56	0.86
Hyperlipidemia, healthy %	42.87	26.22	47.71	0.66
Diabetes, healthy %	85.82	81.27	87.15	0.94
Stroke, healthy %	97.47	96.25	97.82	1.0
Cardiac Arrhythmias, healthy %	82.62	72.66	85.51	1.0
Diastolic BP, [mmHg]	76.54 [10.48]	75.04 [10.07]	76.97 [10.56]	**<0.01** *
Systolic BP, [mmHg]	138.63 [18.44]	141.99 [18.38]	137.65 [18.34]	**<0.01** *
**Genetics**				
*APOE*-ɛ4 SNP (1, 2), %	25.78, 1.57	29.15, 2.69	24.97, 1.30	0.33
*APOE*-ɛ2 SNP (1, 2), %	16.03, 0.96	15.70, 1.35	16.11, 0.86	0.69
*APOE*-ɛ2 PRS, mean [SD]	−0.14 [0.16]	−0.15 [0.17]	−0.14 [0.16]	0.38
*APOE*-ɛ4 PRS, mean [SD]	0.00 [0.30]	−0.02 [0.29]	0.01 [0.29]	0.08
**Lifestyle, mean [SD]**				
PhyAct	9.13 [4.23]	9.01 [4.20]	9.16 [4.24]	0.52
CogAct	20.29 [8.80]	21.13 [8.23]	20.02 [8.96]	**0**.**05**
BMI [kg/m^2^]	28.59 [4.97]	29.26 [5.46]	28.39 [4.80]	**0**.02*

Participants are categorized into amyloid positive (Aβ+) and amyloid negative (Aβ−) based on amyloid PET PIB SUVR cut-point of 1.52 (25 Centiloids). All participants were clinically cognitively unimpaired at baseline. The comparisons shown between the Aβ+ and Aβ− participants are mean comparisons using either Mann Whitney U-test or two-tailed independent *t*-test depending on normality if the variable is continuous or Chi-squared test if the variable is categorical. Statistical significance was set at a *P* < 0.05. The bold values indicate variables with unique *P*-values compared with other variables within their category.

^*^ *P*-value < 0.05.

^a^ln: Natural logarithm of the raw values applied for normalizing the distribution of skewed variables.

ADI, area deprivation index; BCC, body of CC; BMI, body mass index; BP, blood pressure; CAD, coronary artery disease; CC, corpus callosum; CGC, cingulate gyrus part of cingulum; CGH, cingulum hippocampus; CHF, congestive heart failure; CKD, chronic kidney disease; CogAct, cognitive activity; CTh, cortical thickness; FA, fractional anisotropy; GCC, genu of CC; HVA, hippocampal volume adjusted for TIV; PhyAct, physical activity; PRS, polygenic risk score; SNP, Single nucleotide polymorphisms; TIV, total intracranial volume; WMH, white matter hyperintensity fraction volume.

### Overall performance of the models

The baseline cognition prediction models had higher performances (mean *R*^2^ [95% CI]: 0.39 [0.38–0.40]) compared with cognitive decline models (0.27 [0.25–0.28]) (Tables 3 and 4).

**Table 3:** Performance summary of the baseline cognition prediction models

Baseline cognition models					
Models	All	Aβ+	Aβ−	Cohen’s *d*	*P*-value
Socio-demographics	0.35 [0.34–0.36]	0.24 [0.2–0.27]	0.33 [0.31–0.34]	0.96	<0.001*
**Base models**					
Plasma	**0.17 [0.15–0.18]**	−0.03 [−0.06–0.01]	0.16 [0.14–0.19]	1.75	<0.001*
MRI	**0.22 [0.21–0.23]**	0.07 [0.04–0.11]	0.19 [0.16–0.23]	1.35	<0.001*
Cardiovascular	**0.12 [0.11–0.13]**	0.03 [0.01–0.06]	0.10 [0.07–0.13]	1.24	<0.001*
Lifestyle	0.01 [−0.00–0.02]	−0.08 [−0.11 to −0.05]	0.02 [−0.00–0.03]	1.19	<0.001*
Genetics	−0.02 [−0.02 to −0.01]	−0.06 [−0.10 to −0.03]	−0.03 [−0.06 to −0.00]	**0**.**44**	**0.51**
All inputs	0.28 [0.26–0.29]	0.10 [0.07–0.13]	0.27 [0.25–0.28]	1.83	<0.001*
**Base model features + Socio-demographics (Age, sex, education, ADI)**					
Plasma	0.36 [0.35–0.37]	0.24 [0.2–0.27]	**0.36 [0.34–0.39]**	1.05	<0.001*
MRI	0.38 [0.37–0.39]	0.24 [0.21–0.27]	**0.38 [0.35–0.40]**	1.47	<0.001*
Cardiovascular	0.36 [0.35–0.37]	0.25 [0.21–0.28]	**0.36 [0.34–0.38]**	0.95	<0.001*
Lifestyle	0.35 [0.34–0.36]	0.22 [0.18–0.25]	**0.35 [0.33–0.38]**	1.32	<0.001*
Genetics	0.34 [0.33–0.35]	0.22 [0.18–0.26]	**0.34 [0.32–0.37]**	1.01	<0.001*
**Base model features + Socio-demographics + Plasma (Aβ_42_/Aβ_40_, NfL, GFAP, p-tau181)**					
Plasma and MRI	0.39 [0.38–0.40]	0.26 [0.23–0.29]	0.36 [0.35–0.38]	1.36	<0.001*
Plasma and Cardiovascular	0.37 [0.36–0.38]	0.26 [0.23–0.29]	0.35 [0.34–0.37]	1.09	<0.001*
Plasma and Lifestyle	0.36 [0.35–0.37]	0.22 [0.18–0.25]	0.35 [0.34–0.37]	1.44	<0.001*
Plasma and Genetics	0.35 [0.34–0.36]	0.25 [0.22–0.28]	0.33 [0.32–0.35]	0.96	<0.001*
All inputs	**0.39 [0.38–0.40]**	0.29 [0.26–0.31]	0.37 [0.35–0.38]	1.12	<0.001*

The *R*^2^ mean [95% CI] are shown for the cross-validation test folds. The base models consisted of the feature categories only without socio-demographics. The bold values indicate important findings mentioned in Results.

ADI, area deprivation index; GFAP, glial fibrillary acidic protein; NfL, neurofilament light; p-tau181, phosphorylated tau 181.

^*^ *P*-value < 0.05.

**Table 4:** Performance summary of the cognitive decline (slope) prediction models

Cognitive decline (slope) models					
Models	All	Aβ+	Aβ−	Cohen’s *d*	*P*-value
Socio-demographics	0.20 [0.19–0.22]	−0.02 [−0.11–0.06]	0.17 [0.16–0.19]	0.94	0.004*
**Base models**					
Plasma	**0.15 [0.13–0.17]**	0.03 [−0.03–0.09]	0.06 [0.04–0.08]	0.20	0.90
MRI	**0.18 [0.16–0.19]**	0.05 [−0.01–0.1]	0.15 [0.14–0.17]	0.77	0.003*
Cardiovascular	**0.10 [0.08–0.11]**	−0.11 [−0.16 to −0.05]	0.08 [0.06–0.09]	1.35	<0.001*
Lifestyle	−0.01 [−0.01 to −0.01]	−0.15 [−0.2 to −0.1]	−0.03 [−0.04 to −0.02]	0.45	<0.001*
Genetics	−0.02 [−0.03 to −0.01]	−0.16 [−0.22 to −0.1]	−0.02 [−0.03 to −0.01]	0.92	<0.001*
All inputs	0.24 [0.22–0.25]	0.10 [0.05–0.15]	0.17 [0.16–0.18]	0.89	0.08
**Base model features + Socio-demographics (Age, sex, education, ADI)**					
Plasma	**0.23 [0.22–0.25]**	**0.05 [−0.01–0.11]**	**0.17 [0.15–0.19]**	0.76	0.003*
MRI	**0.23 [0.22–0.24]**	**0.09 [0.05–0.14]**	**0.21 [0.19–0.23]**	0.95	<0.001*
Cardiovascular	**0.24 [0.22–0.25]**	0.01 [−0.04–0.06]	**0.19 [0.17–0.20]**	1.27	<0.001*
Lifestyle	**0.19 [0.18–0.21]**	0.06 [0.01–0.11]	**0.17 [0.15–0.19]**	0.78	<0.001*
Genetics	**0.20 [0.18–0.22]**	0.07 [0.01–0.13]	**0.18 [0.16–0.20]**	0.70	0.01*
**Base model features + Socio-demographics + Plasma (Aβ42/Aβ40, NfL, GFAP, p-tau181)**					
Plasma and MRI	**0.26 [0.25–0.28]**	**0.16 [0.11–0.21]**	0.21 [0.20–0.23]	0.40	0.048*
Plasma and Cardiovascular	**0.27 [0.25–0.29]**	0.08 [0.02–0.14]	0.19 [0.18–0.21]	0.72	0.004*
Plasma and Lifestyle	**0.24 [0.22–0.25]**	0.06 [0.01–0.11]	0.17 [0.16–0.19]	0.85	<0.001*
Plasma and Genetics	**0.24 [0.22–0.26]**	0.01 [−0.06–0.08]	0.16 [0.14–0.18]	0.86	0.004*
All inputs	**0.27 [0.25–0.28]**	0.14 [0.10–0.19]	0.20 [0.19–0.22]	0.45	0.03*

The *R*^2^ mean [95% CI] are shown for the cross-validation test folds. The base models consisted of the feature categories only without socio-demographics. The bold values indicate important findings mentioned in Results.

ADI, area deprivation index; GFAP, glial fibrillary acidic protein; NfL, neurofilament light; p-tau181, phosphorylated tau 181.

^*^ *P*-value < 0.05.

**Figure 2:**
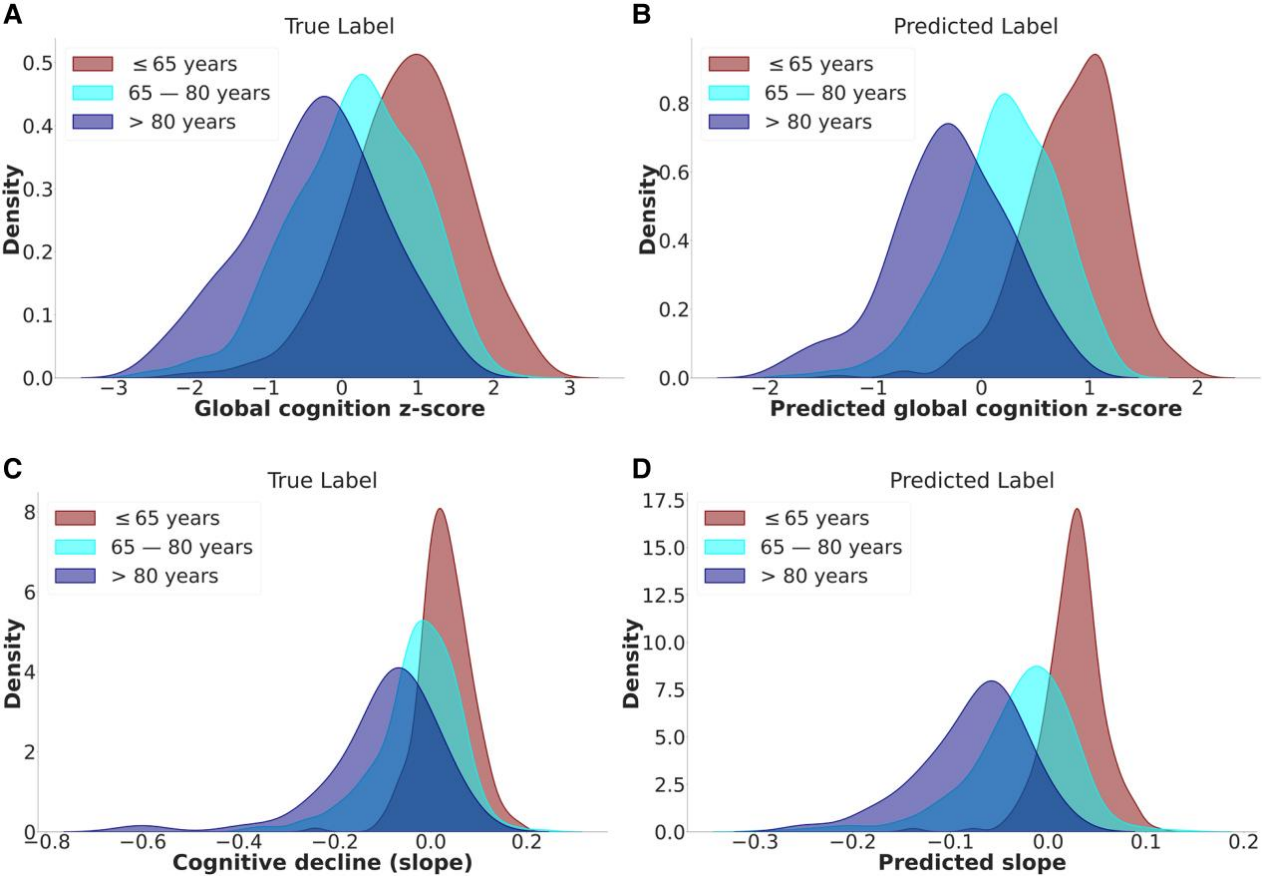
**Model predictions.** Comparison of the histogram of the true and predicted baseline cognition (**A** and **B**) and cognitive decline (slope) (**C** and **D**) models arranged by age strata.

The baseline cognition and cognition decline models were able to capture the age-related trend of cognition across the age-strata (Fig. 2).

### Plasma biomarkers versus other predictors in the base models

**Figure 3:**
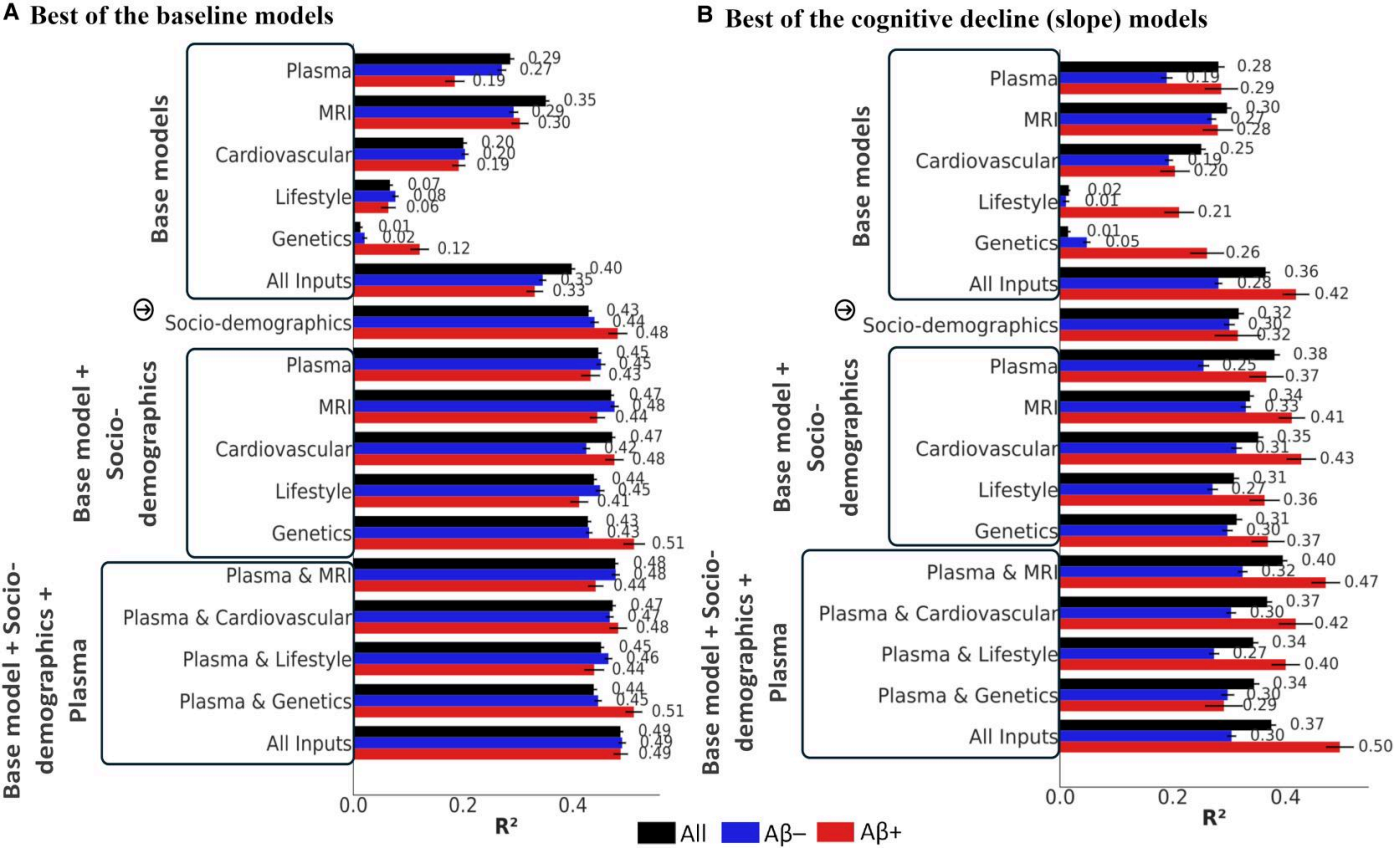
**Performances of the best baseline (A) and cognitive decline models (B).** The error bars are the standard error of mean for the cross-validated runs with the value of the best *R*^2^ shown at the end of each bar. The base models included only the predictor variables within a biomarker category. The arrow shows the socio-demographics model (age, sex, education, and area deprivation index). We defined ‘base’ as the exclusion of socio-demographics and inclusion of only the biomarkers, and the advanced models progressed with socio-demographics first added and then plasma and socio-demographics added to the base models. Aβ, beta amyloid.

The base model using plasma biomarkers alone yielded a mean *R*² of 0.17 [95% CI: 0.15–0.18] and 0.15 [0.13–0.17] for the baseline and cognitive decline models (Tables 3 and 4). This was comparable to MRI and cardiovascular models which demonstrated a mean *R*² of 0.22 [0.21–0.23] and 0.18 [0.16–0.19], and 0.12 [0.11–0.13] and 0.10 [0.08–0.11], respectively for the baseline and cognitive decline models. Both lifestyle factors and genetics showed lower performances compared with the other predictors with *R*² close to zero for both baseline and cognitive decline models (Tables 3 and 4). The best performing base models for plasma (*R*^2^ baseline, *R*^2^ decline: 0.29, 0.28), MRI (0.35, 0.30), cardiovascular (0.20, 0.25), lifestyle (0.07, 0.02) and genetics (0.08, 0.01) showed a similar trend between their baseline and cognitive decline model counterparts (Fig. 3).

### Addition of socio-demographics and plasma biomarkers to base models

We observed improvements when socio-demographics were included to the base models in both baseline and cognitive decline models (Tables 3 and 4). The baseline models showed a greater and consistent improvement compared with the decline models. Further combining socio-demographics and plasma with other markers slightly improved the models’ performance. For instance, in the cognitive decline models the combination of socio-demographics, plasma and MRI yielded a mean *R*² of 0.26 [95% CI: 0.25–0.28], indicating that plasma biomarkers, when used alongside structural brain MRI measures, may offer slightly more predictive value over plasma biomarkers or MRI measures alone.

Integrating plasma biomarkers with cardiovascular and socio-demographics data also showed improved prediction of cognitive decline with a mean *R*² of 0.27 [0.25–0.29], akin to the effect observed with brain health MRI measures. Similarly, the lifestyle and genetics models with the addition of plasma and socio-demographics produced *R*² means of 0.24 [0.22–0.25] and 0.24 [0.22–0.26], respectively, suggesting that the addition of plasma biomarkers to these models enhanced predictive accuracy in a similar manner to the brain health MRI and cardiovascular measures (Table 4).

### Differential performance of models based on amyloid status

In the baseline cognition models stratified by amyloid status, Aβ− models generally significantly outperformed Aβ+ models across cross-validated runs (*P* < 0.05) except for the genetics models (*P* = 0.51, Cohen’s *d* = 0.44) (Table 3). However, the best Aβ+ genetics models consistently outperformed Aβ− models (base model: *R*^2^ = 0.12 versus 0.02, base + socio-demographics model: *R*^2^ = 0.51 versus 0.43, base + socio-demographics + plasma model: *R*^2^ = 0.51 versus 0.45) (Fig. 3).

In the cognitive decline models, the mean *R*^2^ of the cross-validated Aβ− models was significantly greater than their Aβ+ counterparts (Table 4). However, notably, the Aβ+ models showed marked improvement when plasma was combined with MRI measures (plasma + socio-demographics models: *R*^2^ = 0.05 [−0.01–0.11], MRI + socio-demographics models *R*^2^: 0.09 [0.05–0.14], combined models: *R*^2^ = 0.16 [0.11–0.21]) (Table 4). The best Aβ+ base cognitive decline models showed higher performance in predicting cognitive decline compared with Aβ− models (Fig. 3).

### ML models revealed heterogeneity of predictors of cognition across age groups

Characteristics tables for the age groups described below can be found in Supplementary Tables 1–3.

#### Younger age group (<65 years)

**Figure 4:**
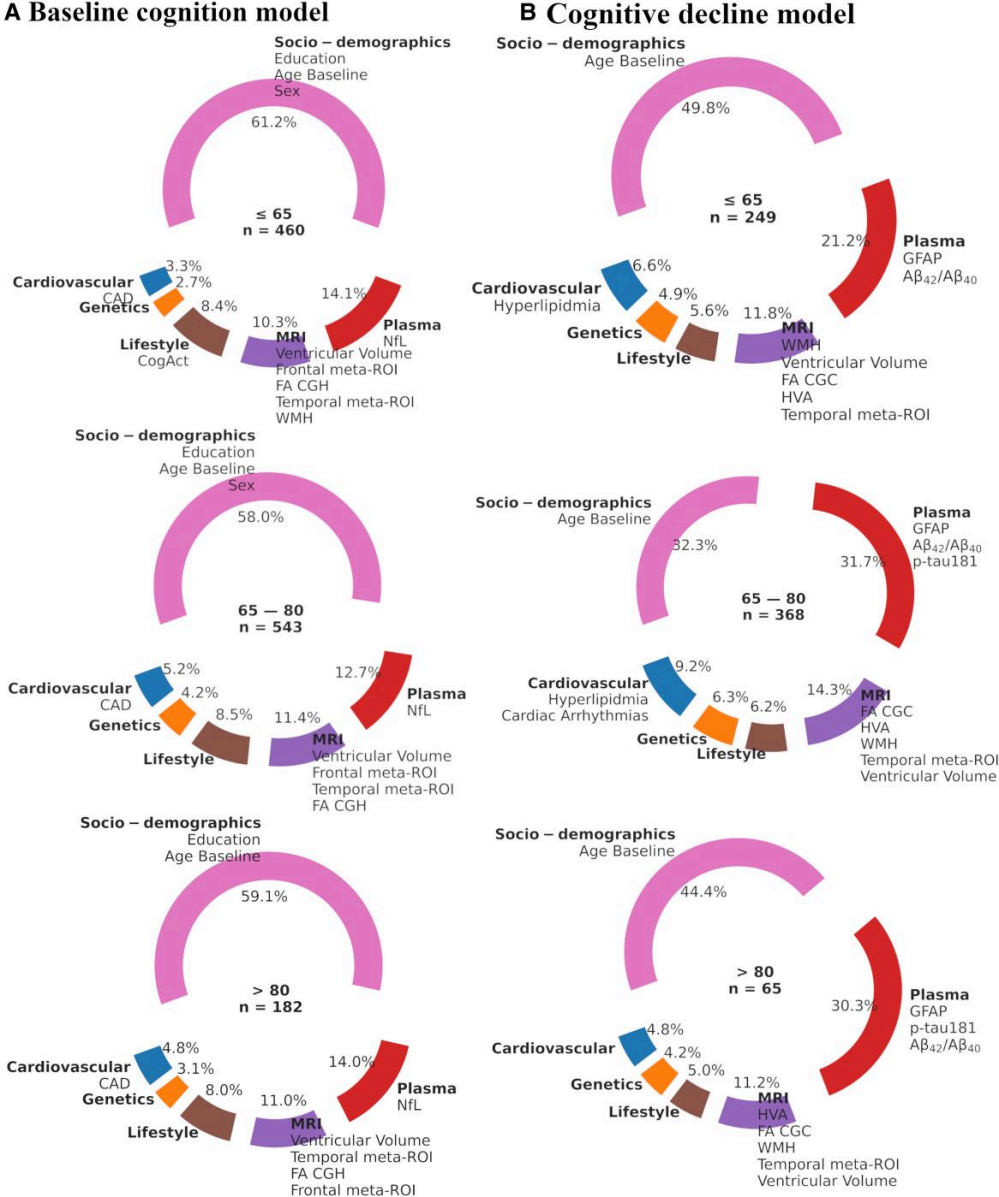
**Feature importance across the age strata for the baseline (A) and cognitive decline models (B).** All participants were cognitively unimpaired, and the pie charts indicate the contribution of the predictors within each age group, shown at their centers in years. The number of participants is given as *n* for each group. The top features are listed under each category. Aβ, beta amyloid; CAD, coronary artery disease; CGC, cingulate gyrus part of cingulum; CGH, cingulum hippocampus; CogAct, cognitive activity; FA, fractional anisotropy; GFAP, glial fibrillary acidic protein; HVA, hippocampal volume; ND, Neurodegeneration; NfL, neurofilament light; p-tau181, phosphorylated tau 181.

In the group with younger individuals under 65 years, NfL was the top plasma predictor for the baseline models (*n* = 460) while GFAP and Aβ_42_/Aβ_40_ ratios were predominant in the decline models (*n* = 249) (Fig. 4). In addition to a range of structural MRI measures, ventricular volume and temporal meta-ROI (ND Marker) were top predictors in both baseline and decline models (Fig. 4).

**Figure 5:**
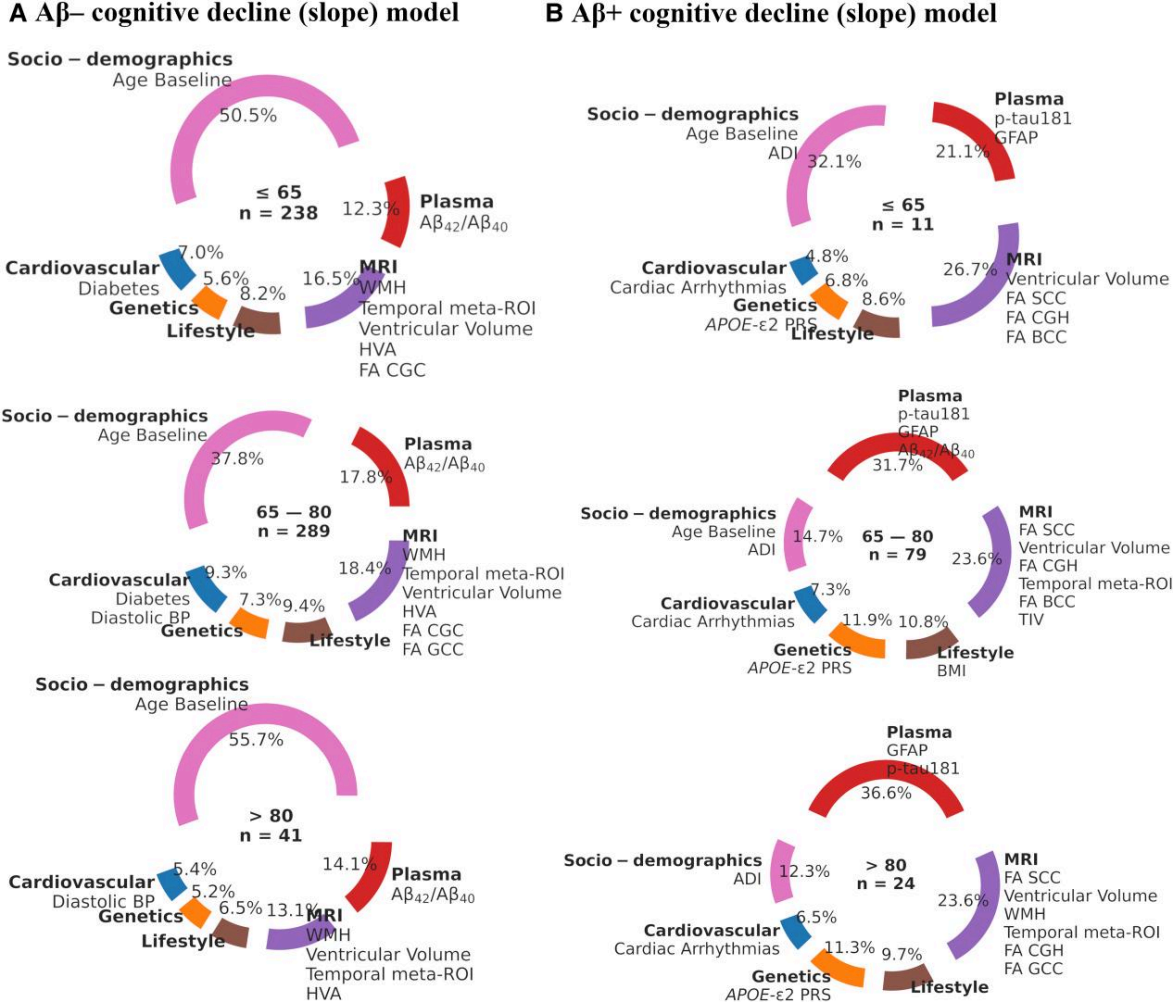
**Feature importance across the age strata for cognitive decline prediction models based on amyloid status.** The pie charts show the contribution of the predictors for Aβ+ (**A**) and Aβ− (**B**) models across the age groups, shown at their centers in years. The number of participants is given as *n* for each group. The top features are listed under each feature category. Similar feature importance summaries of the baseline model are shown in Supplementary Fig. 3. Aβ, beta amyloid; ADI, area deprivation index; *APOE*, apolipoprotein E; BCC, body of CC; BMI, body mass index; CC, corpus callosum; CGC, cingulate gyrus part of cingulum; CGH, cingulum hippocampus; FA, fractional anisotropy; GCC, genu of CC; GFAP, glial fibrillary acidic protein; HVA, hippocampal volume; ND, neurodegeneration; p-tau181, phosphorylated tau 181; PRS, polygenic risk score; TIV, total intracranial volume; WMH, white matter hyperintensity fraction volume.

When looking at the decline models based on amyloid status, the Aβ− decline models (*n* = 238) were predicted by plasma Aβ_42_/Aβ_40_, MRI ventricular volume, temporal meta-ROI (ND Marker), WHM, HVA and FA CGC, and cardiovascular diabetes (Fig. 5). In contrast, the Aβ+ decline models (*n* = 11) were uniquely predicted by plasma p-tau181 and GFAP, FA SCC, FA CGH, FA BCC, genetics *APOE*-ԑ2 PRS, cardiovascular cardiac arrhythmias and the socio-demographics measures age and ADI (Fig. 5).

#### Middle age group (65–80 years)

In the 65 to 80-year age group, plasma NfL was one of the top predictors for the baseline models (*n* = 543). In contrast, Aβ_42_/Aβ_40_, GFAP and p-tau181 were predominant predictors in the decline models (*n* = 368) (Fig. 4).

When delineated by amyloid status, plasma p-tau181 and GFAP were key predictors in the Aβ+ decline model (*n* = 79) while plasma Aβ_42_/Aβ_40_ was the top plasma predictor in both models (Aβ− *n* = 289) (Fig. 5). The MRI markers, which were relevant in the younger age groups, in general continued to be relevant in the middle-aged group, with the notable exception of FA GCC in the Aβ− baseline models and TIV in the Aβ+ decline models (Fig. 5).

#### Older age group (>80 years)

In individuals older than 80 years, NfL was a key predictor in the baseline model (*n* = 182) while GFAP, p-tau181, and Aβ_42_/Aβ_40_ were the top predictors in cognitive decline model (*n* = 65). The MRI predictors for this age group were very similar to those identified in the younger and middle-aged groups (Fig. 4).

When looking at the top features based on amyloid status, plasma Aβ_42_/Aβ_40_ was the main predictor in the Aβ− decline models (*n* = 41) while GFAP and p-tau181 were primary predictors in the Aβ+ decline model (*n* = 24) (Fig. 5). In the Aβ+ decline model for this age group, MRI TIV was no longer a top predictor, whereas FA GCC transitioned from being a top predictor in the Aβ− models to the Aβ+ models (Fig. 5).

## Discussion

In this study, we present a ML approach for predicting cognition in a cohort of cognitively unimpaired participants by using baseline measures of plasma, brain health, cardiovascular, genetics, and lifestyle. Given the increasing interest in the development and utility of plasma biomarkers in aging and dementia studies, we focused on evaluating their effectiveness when modeled separately and in combination with other known factors for predicting cross-sectional cognition as well as longitudinal cognitive decline. We designed a set of experiments to uncover scenarios where plasma biomarkers (Aβ_42_/Aβ_40_, NfL, GFAP, p-tau181) would be most useful compared with brain health MRI (structural and diffusion), cardiovascular, genetics and lifestyle measures.

###  

#### Major findings

1. Plasma biomarkers, brain health and cardiovascular measures were similarly predictive of cognitive decline and performed better compared with the genetics and lifestyle.
  1.1. With socio-demographics included in the models, all measures explained similar variance in predicting cognitive decline.
2. If an individual was amyloid negative, socio-demographics were the most useful predictor and explained most of the variance in predicting cognitive decline.
  2.1. Aβ_42_/Aβ_40_ was the top plasma feature for predicting cognitive decline across all age groups, and its contribution increased from younger to mid age group and decreased to the older age group.
3. In amyloid positive individuals, plasma biomarkers explained most of the variance in predicting cognitive decline.
  3.1. p-tau181 and GFAP were the most important plasma predictors for predicting cognitive decline and their contribution increased with age.
4. No single biomarker category explained more than 50% of the variance in any of the cognitive decline models. The highest *R*^2^ values when predicting cognitive decline were observed when all measures were combined (mean *R*^2^ = 0.27, best *R*^2^ = 0.50). This low explanatory power highlights the limited clinical utility and diagnostic value of the individual biomarkers for cognitive decline prediction.

A novel aspect of our work involved incorporating a comprehensive list of known risk factors alongside plasma biomarkers to predict cognitive outcomes. Recently, several studies have been published that highlight plasma biomarkers as predictors of cognition, predictors of clinical AD dementia, and predictors of AD conversion in a CU or mild cognitive impairment (MCI) cohort. For instance, Palmqvist *et al*. reported combining plasma p-tau217 with measurement of memory and executive function showed higher accuracy in predicting AD clinical diagnosis within 4 years.^46^ Stevenson-Hoare *et al*. predicted AD clinical diagnosis by combining Aβ_40_ or Aβ_42_, p-tau217, GFAP, NfL, *APOE*-ԑ4 and PRS and obtained an area under the curve (AUC) of 0.81.^47^ Binette *et al*. reported in an MCI population combination of p-tau217, HVA, *APOE* genotype, and cognition as predictors were able to discriminate progression to AD dementia at AUC of 0.89.^48^

#### Combining plasma biomarkers with other predictors improved performance

Initially, we employed models excluding socio-demographic variables to isolate the impact of various predictors on cognitive decline. This approach allowed us to assess their predictive value independently. We then evaluated their combined effect with plasma biomarkers and other categories on the models’ performances. We found that when amyloid status was not considered the plasma biomarkers contributed 21.2%, 31.7% and 30.3% of top features that explained the variance in predicting cognitive decline for individuals aged younger than 65 years, between 65 and 80 years and older than 80 years, respectively (Fig. 4). In both baseline and cognitive decline models, socio-demographics were prominent, yet their relative contributions varied. Specifically, socio-demographics accounted for a larger share of variance (>50%) in baseline models (Fig. 4). When socio-demographics were paired with plasma biomarkers, there were varying degrees of improvement in models’ predictive performance for cognitive decline, suggesting that their role could be additive interaction in the presence of other factors. Overall, the integration of plasma biomarkers with socio-demographics and other biomarkers augmented the predictive accuracy of the cognitive decline best models.

#### Not all plasma biomarkers were equally important across age groups and amyloid status

Our results suggest that plasma biomarkers may reflect differential temporal processes in the AD pathophysiologic cascade, in line with previously published findings. A recent study on Aβ+ individuals (mean age 73 years) by Mattsson-Carlgren *et al*. found p-tau217 to be the plasma biomarker most associated with longitudinal cognitive decline.^49^ Another study by Cullen *et al*. found that Aβ_42_/Aβ_40_ relates to early changes (A), p-tau217 to late changes (T), and NfL to neurodegeneration (N) in the ATN scheme.^6^ We also found p-tau181 to be an important predictor of cognitive decline most relevant for older individuals (>65 years), whereas for younger individuals (<65 years) GFAP and Aβ_42_/Aβ_40_ were the most important biomarkers (Fig. 4). However, GFAP was found to be a top predictor across all age strata in cognitive decline models and especially for Aβ+s. In addition, for Aβ− individuals, Aβ_42_/Aβ_40_ was the most important biomarker when predicting cognitive decline (Fig. 5).

#### Differences in predictive ability of genetics and lifestyle

The predictive capacity of genetic and lifestyle factors, although lower than that of plasma, MRI and cardiovascular measures, still provided important predictive value for both baseline cognition and cognitive decline models. The contribution of genetics to predict cognitive decline showed higher contribution in the Aβ+ individuals from 6.8 to 11.3% (Fig. 5). Notably, in these models, *APOE*-ԑ2 PRS was the most important predictor where higher *APOE-*ԑ2 risk scores indicated a faster cognitive decline (Supplementary Fig. 4). There is some evidence to suggest PRSs could account for unique disease heterogeneity when incorporated with plasma biomarker^26^ as well as predict cognitive decline.^50^ On the contrary, lifestyle’s largest contribution was in the baseline Aβ− models (>11.0%) (Supplementary Fig. 3) where increasing cognitive activity and light physical activity were indicative of better baseline cognition (Supplementary Fig. 4). Although, previous studies suggest light midlife physical activity or vigorous late-life physical activity being associated with lesser decline in cognition,^24^ we did not find lifestyle to have a strong influence in cognitive decline models. Nonetheless, quantifying the relationship between lifestyle and cognitive decline is a challenge due to differences in individual lifestyles, such as diet, alcohol use, smoking, leisure activities and education, compounded by underlying genetic risk factors and cardiovascular comorbidities.^51,52^

#### Strengths and limitations

A strength of this study was the inclusion of a wide spectrum of variables to uncover the role of plasma biomarkers in predicting cognitive decline. There were also some limitations. First, prior exposure to cognitive tests was not included in the modeling. It was excluded after conducting validation analyses which showed potential confounding interaction with other predictors and the targets. A linear regression analysis found prior exposure positively associated with baseline cognitive scores (*β* = 0.07, *P* < 0.001), but negatively associated with cognitive decline values (*β* = −0.015, *P* < 0.001) suggesting an initial practice effect followed by a decline in cognition (Supplementary Table 4). In addition, when we included it in the ML models, we observed it dominated other predictors due to its strong interaction with the cognition outcomes (Supplementary Tables 5 and 6, Supplementary Fig. 5). Taken together, prior exposure was found to be a confounding variable having strong associations with the predictor and target variables with potential to bias model interpretations warranting its exclusion from modeling. Second, the plasma analytes were measured using the Quanterix Simoa which relies on the single molecule enzyme-linked immunosorbent assay (ELISA). However, numerous studies have demonstrated variations among different ELISA assay types, and these also differ from other assay methods, such as mass spectrometry.^10,14,22,27,36^ Further studies are needed to confirm and compare the results with additional biomarkers as well as a replication of the results with biomarkers obtained using mass spectrometry.

## Conclusion

The primary aim of our study was to investigate the diagnostic value of plasma biomarkers to predict cognitive outcomes in the presence of various known risk factors and biomarkers. Through a set of experiments, we identified scenarios where plasma biomarkers were significantly better at predicting cognition and where their usefulness was lower. Overall, plasma biomarkers’ performance was in line with brain health and cardiovascular measures. The variance explained with and without plasma biomarkers present in the models was similar indicating although they have slight confounding effects, they seem to provide complementary information for prediction of cognitive outcomes.

## Supplementary Material

fcae300_Supplementary_Data

## Data Availability

Mayo Clinic Study of Aging data are available to qualified academic and industry researchers by request to the MCSA and ADRC Executive Committee (https://www.mayo.edu/research/centers-programs/alzheimers-disease-research-center/research-activities/mayo-clinicstudy-aging/for-researchers/data-sharing-resources). Codes are available at GitHub: https://github.com/RobelGebre/CognitionPredictionWithPlasma.
