# Supplementary material for: Can integration of Alzheimer’s plasma biomarkers with MRI, cardiovascular, genetics, and lifestyle measures improve cognition prediction?
Source: Brain Commun. 2024 Sep 4;6(5):fcae300. doi: 10.1093/braincomms/fcae300 (PMC11406552; doi:10.1093/braincomms/fcae300)
Supplement: fcae300_Supplementary_Data [file fcae300_supplementary_data.docx]

#
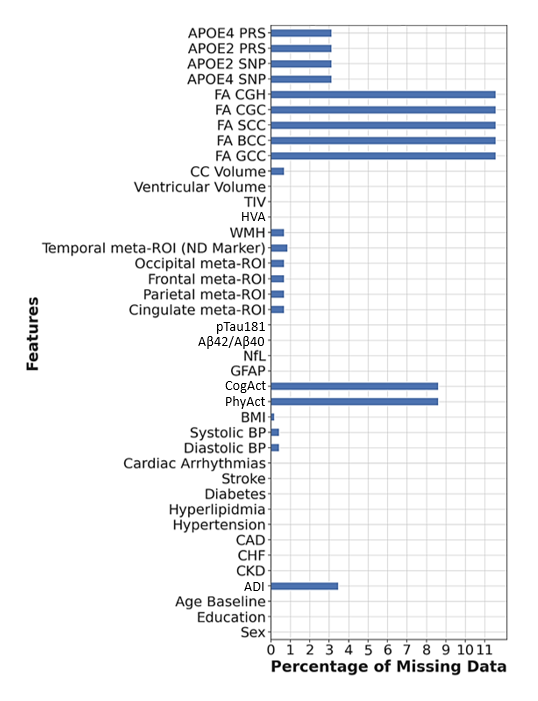
Supplementary Figures

APOE = apolipoprotein E, SNP = Single nucleotide polymorphisms , PRS = Polygenic risk score, FA = Fractional anisotropy, BCC = Body of CC, CGC = cingulate gyrus part of cingulum, CGH = Cingulum hippocampus, GCC = Genu of CC, CC = Corpus callosum, WMH = White matter hyperintensity fraction volume, HVA = Hippocampal volume adjusted for TIV, TIV = Total intracranial volume, ND = Neurodegeneration, CKD = Chronic kidney disease, CHF = Congestive heart failure, CAD = Coronary artery disease, BP = Blood pressure, PhyAct = Physical activity, CogAct = Cognitive activity, ADI = Area Deprivation Index National Rank, BMI = Body mass index, GFAP = Glial fibrillary acidic protein, NfL = Neurofilament light, pTau181 = Phosphorylated tau, ADI = Area Deprivation Index National Rank.

**Supplementary Figure 1**. Summary of missing data.


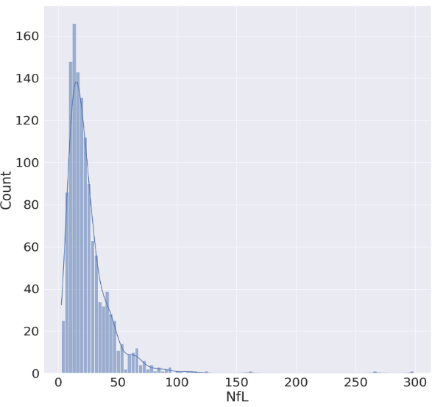

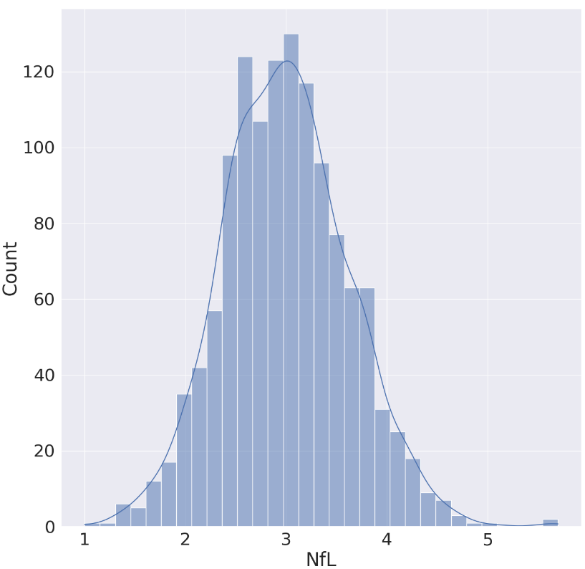

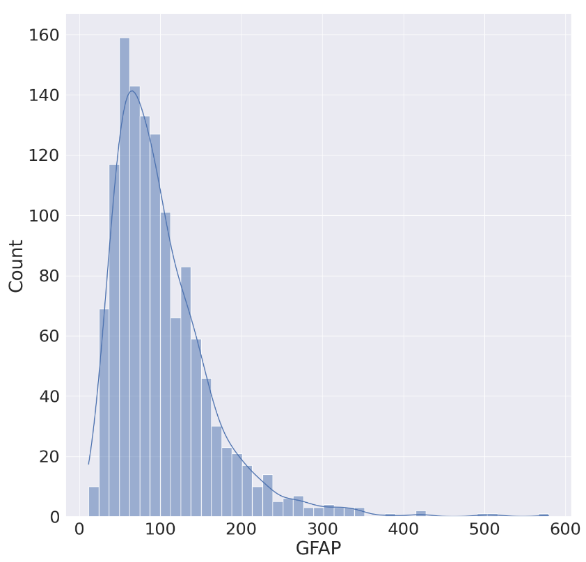

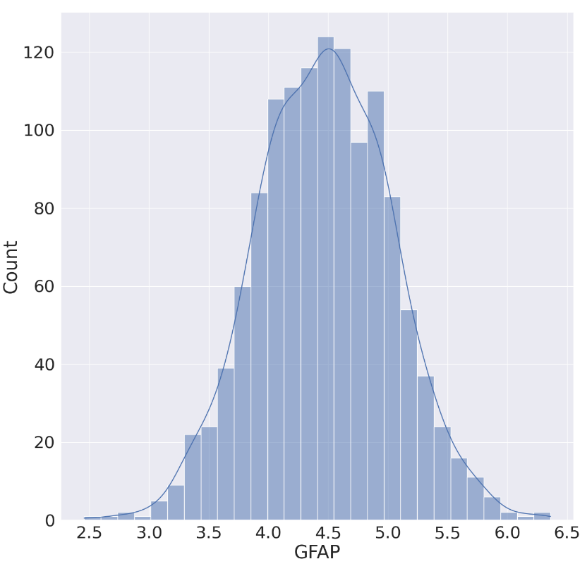

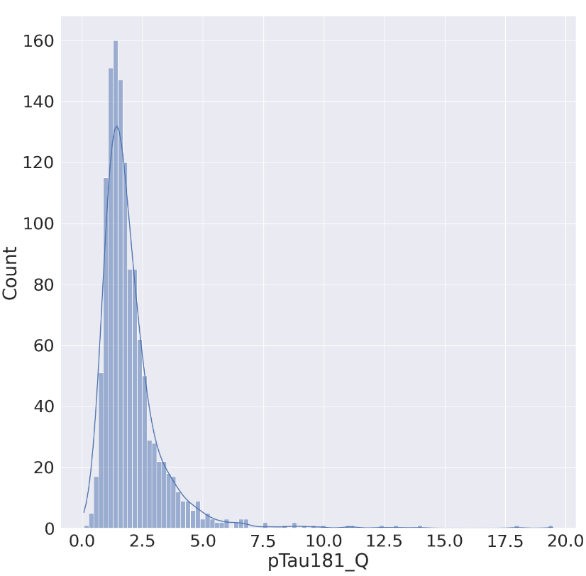

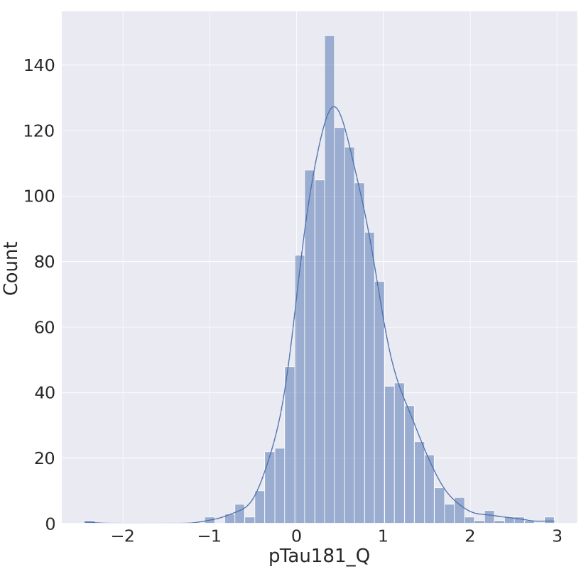

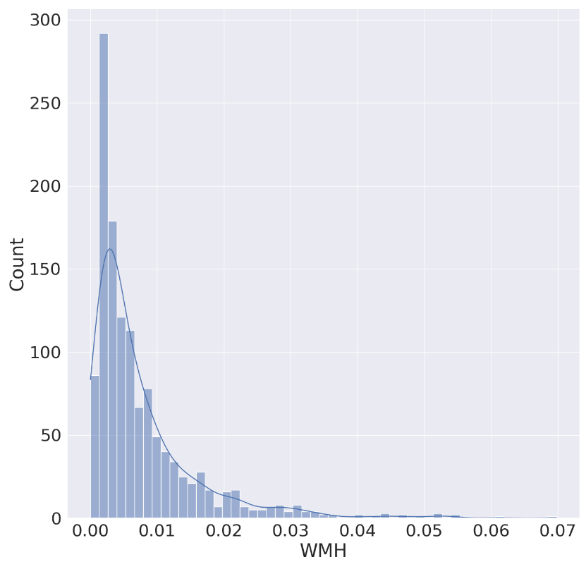

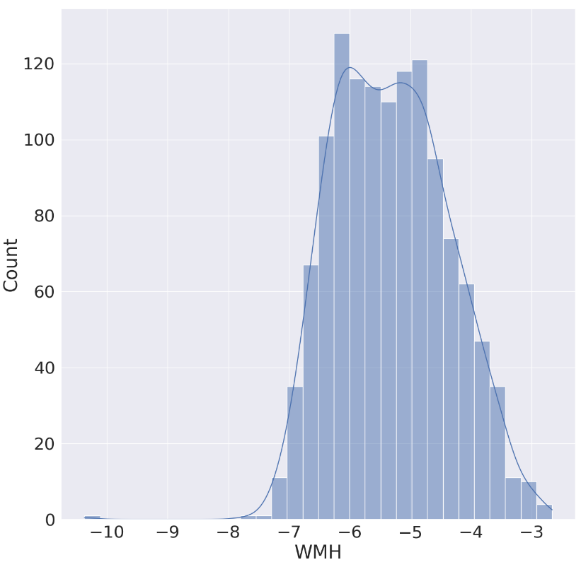


**Supplementary Figure 2**. Normalization preprocessing on the inputs that were skewed. The natural logarithm was taken in each case to normalize their distributions. The arrows indicate the transformation before and after taking the natural logarithm.


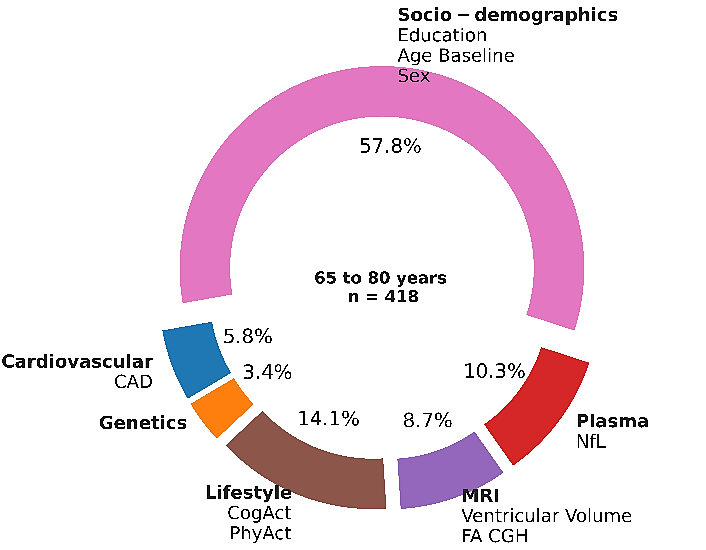

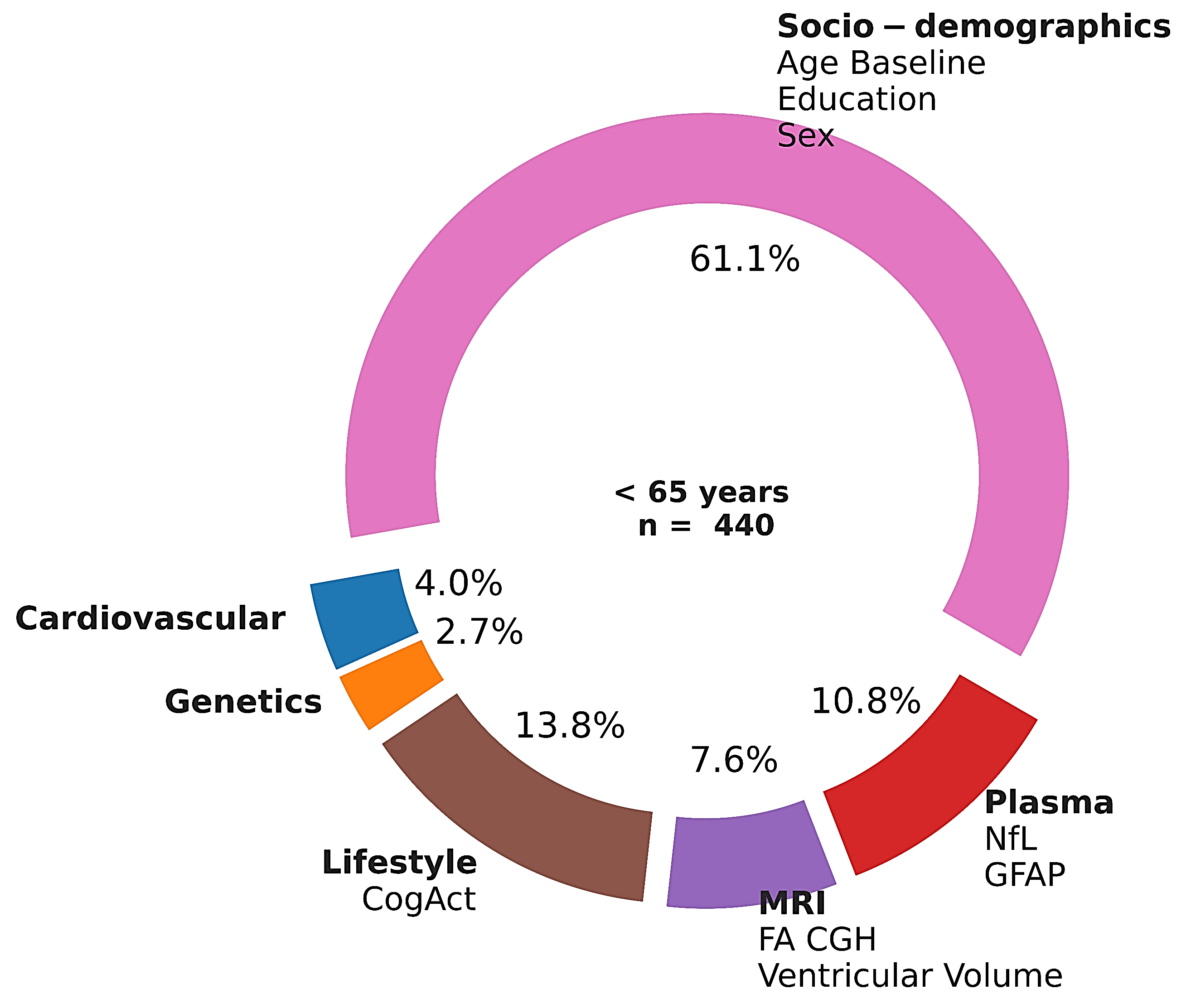


**Aβ– Baseline Model**


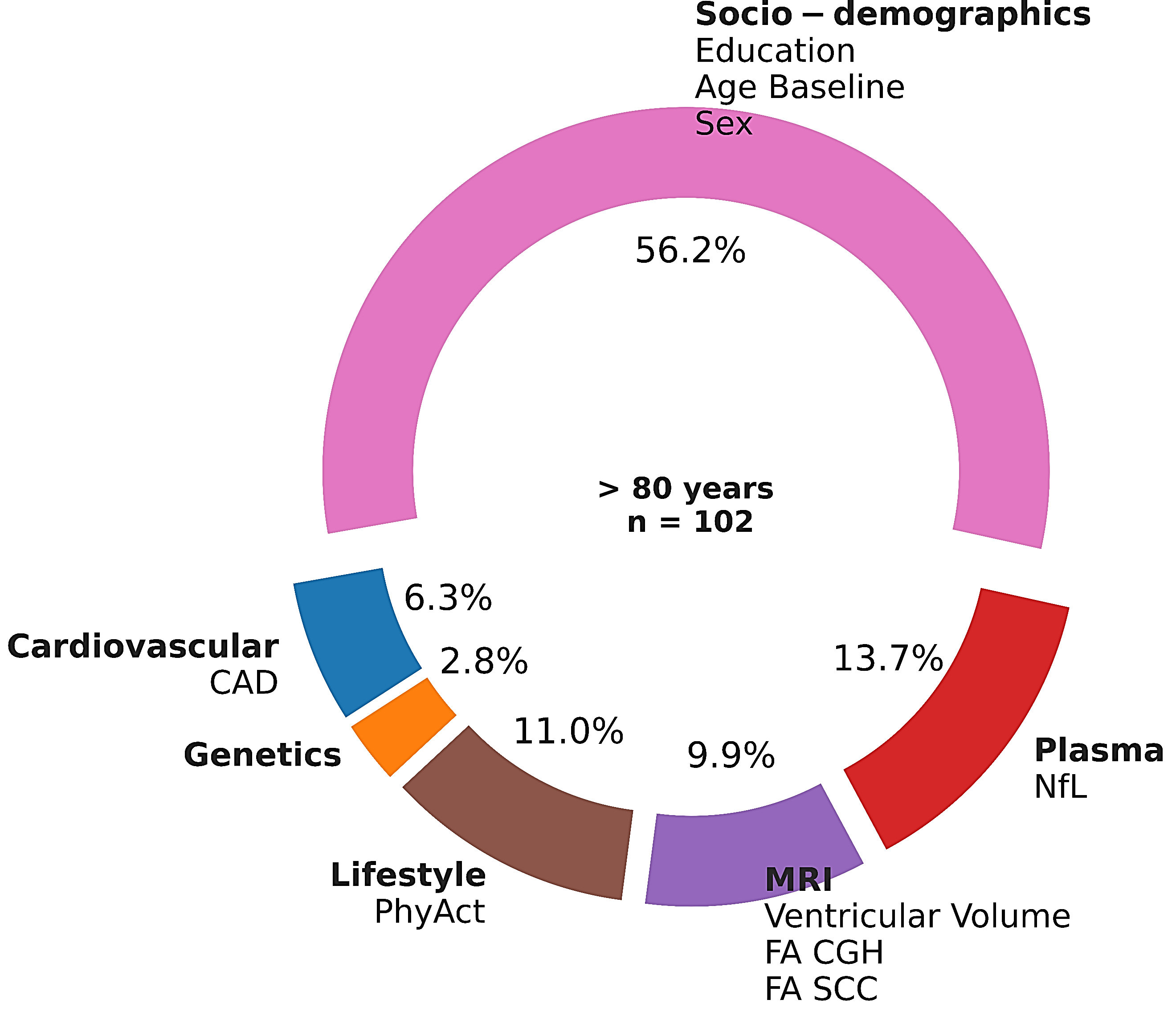

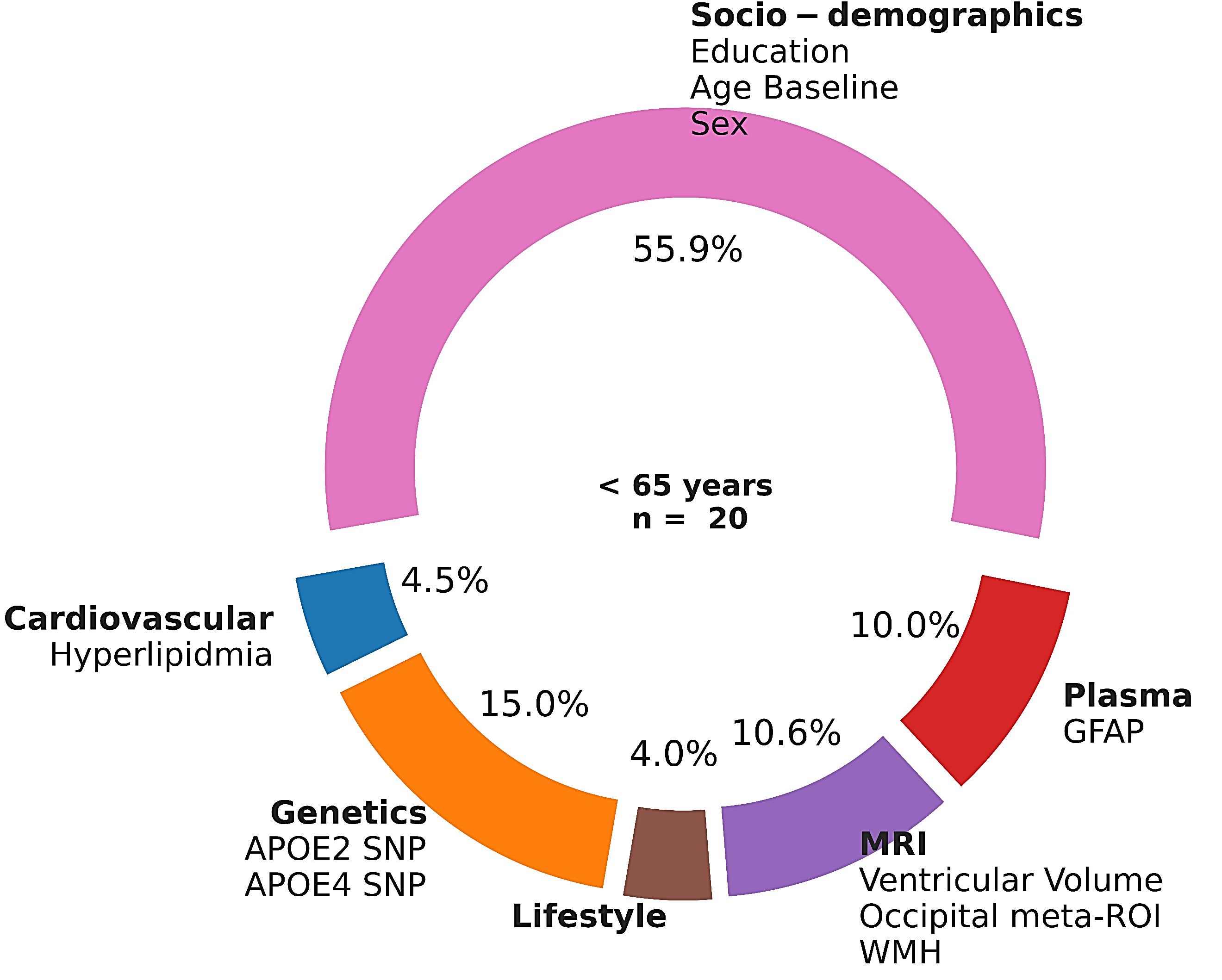

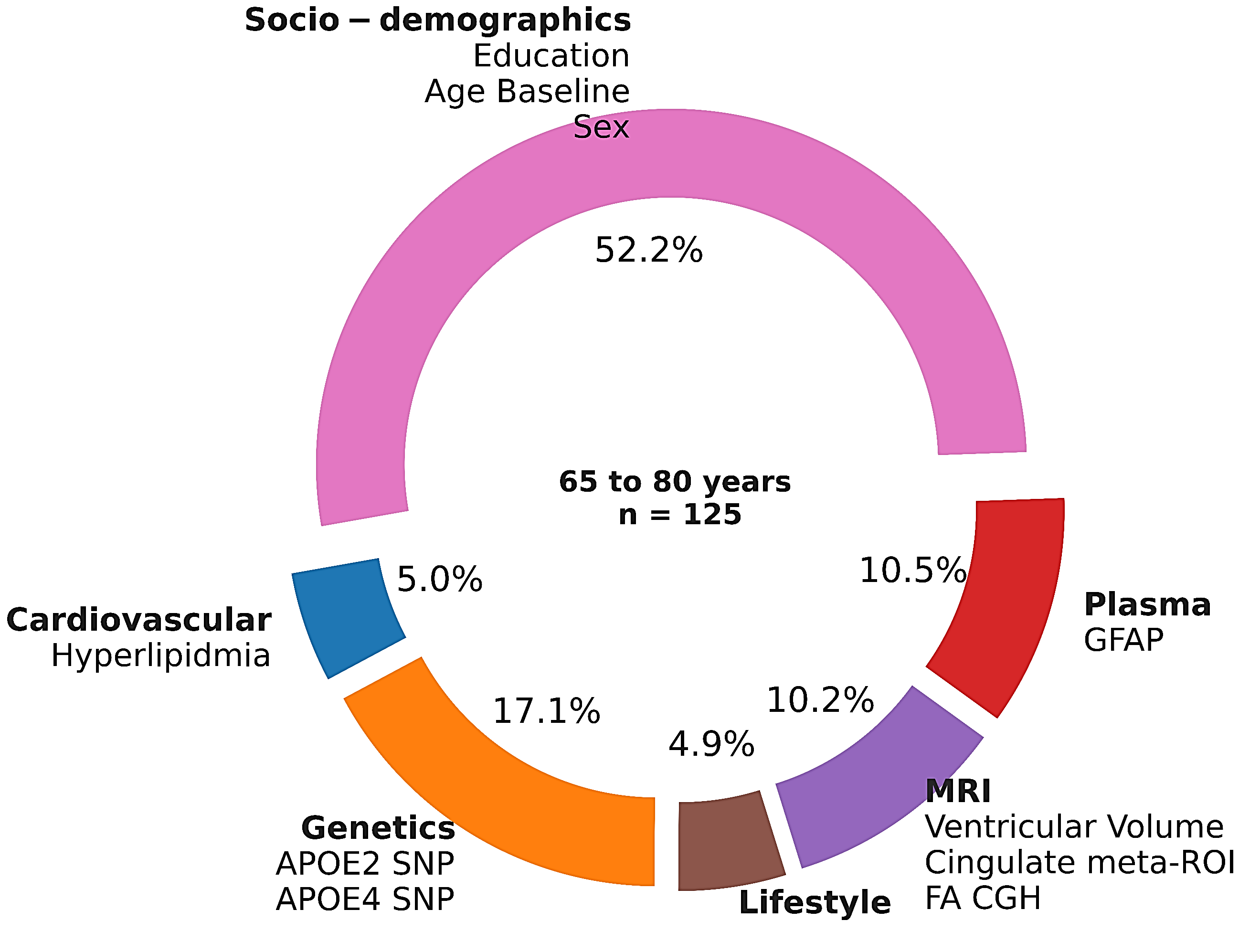


**Aβ+ Baseline Model**


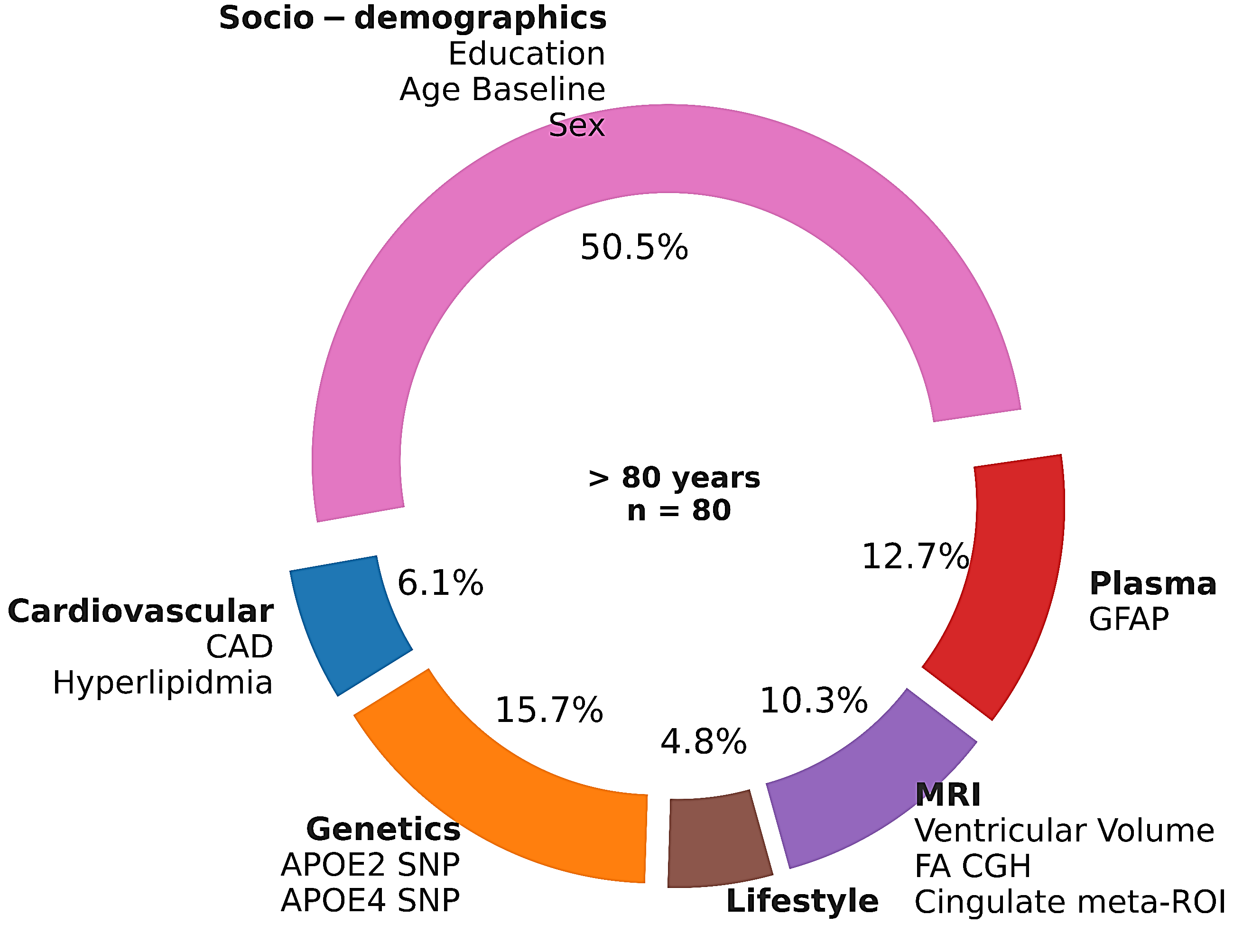


**Supplementary Figure 3.** Feature importance across the age strata for baseline cognition prediction models. The pie charts show the contribution of a feature category based on its importance and the top features are listed under each category.

**Supplementary Figure 4.** The relationship between APOE2 PRS, cognitive and physical activities to their respective model contributions (i.e., SHAP values). APOE2 has a somewhat linear trend, with higher risk score corresponding to faster decline. Cognitive and physical activity are inversely related to baseline cognition prediction. Code used to fit the curves and identify the transition points can be found here <https://github.com/RobelGebre/TPE-for-SHAP>

**Cognitive Activity**

**SHAP values for Cognitive Activity**


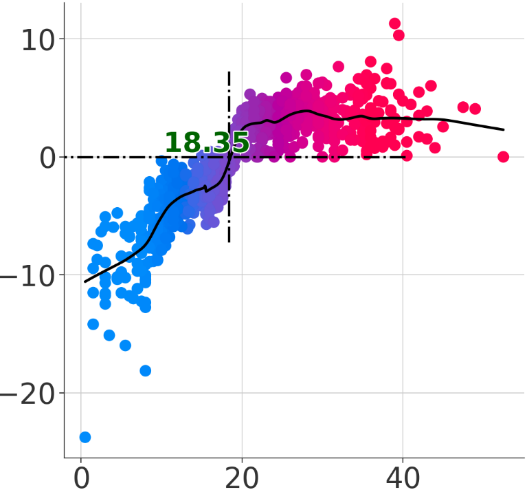

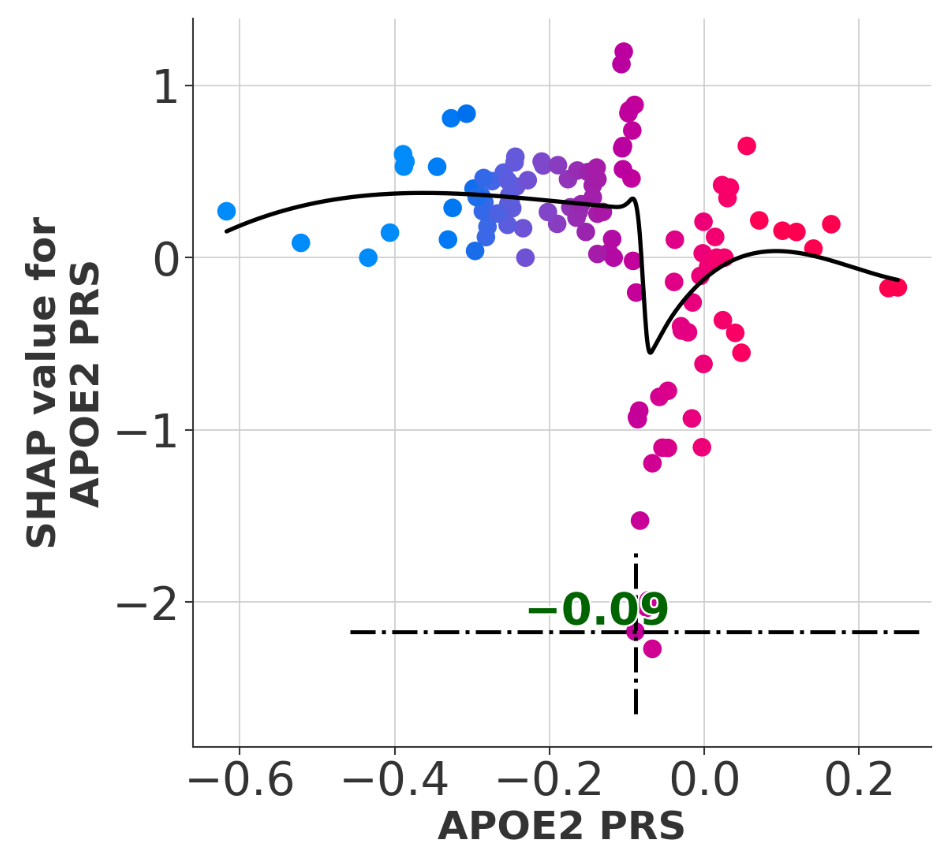


**Physical Activity**

**SHAP values for Physical Activity**


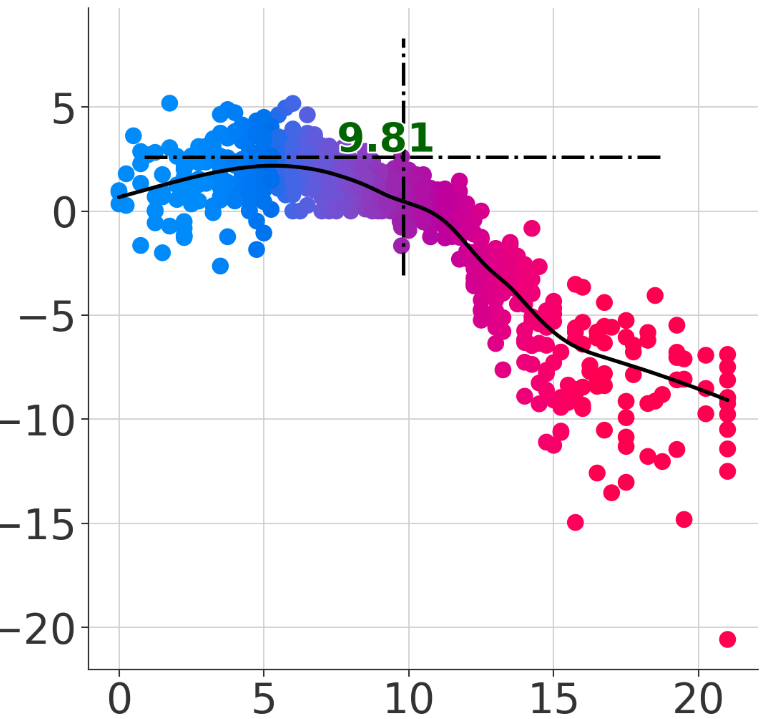


**Aβ– baseline**

**Aβ– baseline**

**Aβ+ decline**


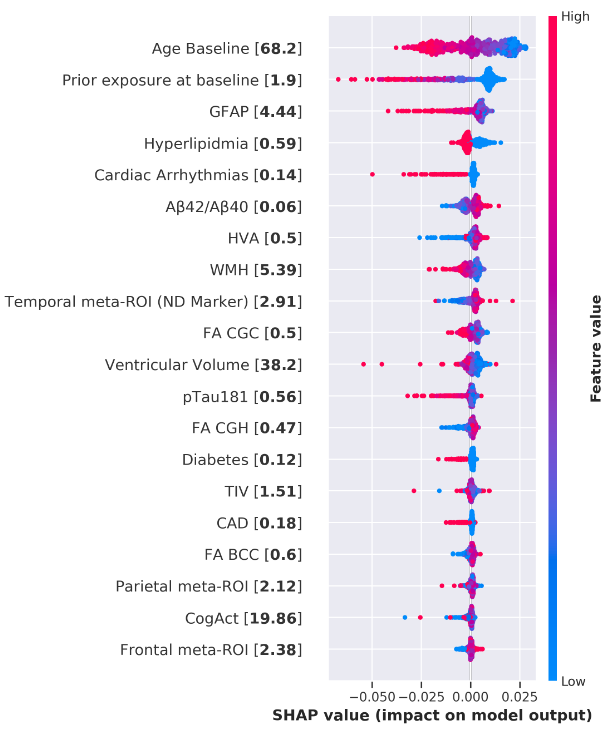

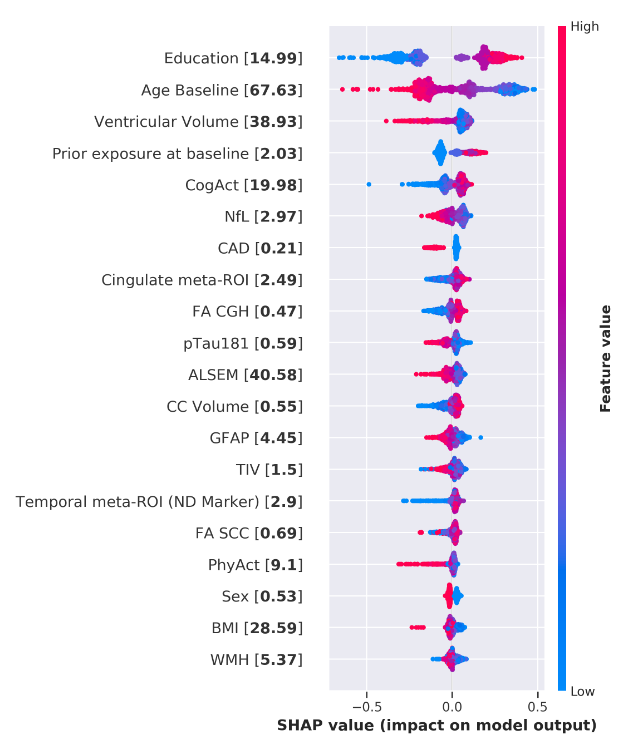


**Supplementary Figure 5.** Feature importance for (A) baseline and (B) decline models to demonstrate behavior of prior exposure in the cognition prediction models. The results agree with the linear regression results shown in Supplementary Table 4 below: a greater number of exposures at baseline were indicative of better cognition which was not was not protective in the long-term.

**(A) Baseline Models**

**(B) Decline Models**

# Supplementary Tables

**Supplementary Table 1.** Summary table for participants younger than 65 years old used in the baseline and decline models. The Baseline and Decline columns show the characteristics of the same participant measured at baseline, however, for the decline models those with exactly 5 follow-ups from baseline were included, hence the reduced number of participants in the Decline column.

|  | **Baseline** | | | | **Decline** | | |
| --- | --- | --- | --- | --- | --- | --- | --- |
|  | **Aβ+** | **Aβ –** | ***P-value*** | **Aβ+** | | **Aβ –** | ***P-value*** |
| Number of participants, *n* | 20 | 440 |  | 11 | | 238 |  |
| Cognition z-scores, *mean [SD]* | 0.66 [0.82] | 0.88 [0.77] | 0.12 | 0.78 [0.97] | | 1.11 [0.78] | 0.21 |
| Sex, *male* *%* | 30% | 50% | 0.72 | 36% | | 55% | 0.22 |
| Age, y, mean [SD] | 61.58 [4.08] | 55.10 [8.53] | < 0.001 | 60.71[4.29 | | 58.00 [5.6] | 0.08 |
| Practice effect, mean [SD] | 1.35 [0.59] | 1.18 [0.48] | 0.08 | 1.18 [0.40] | | 1.17 [0.43] | 0.81 |
| Education, *mean [SD]* | 15.10 [2.05] | 15.48 [2.22] | 0.43 | 14.45 [1.75] | | 15.43 [2.13] | 0.16 |
| ADI, *mean [SD]* | 40.0 [19.40] | 37.39 [15.86] | 0.75 | 36.91 [13.77] | | 37.94 [15.62] | 0.99 |
| $\ln GFAP$ | 4.37 [0.42] | 4.05 [0.44] | < 0.001 | 4.35 [0.38] | | 4.11 [0.40] | 0.053 |
| $\ln NfL$ | 2.75 [0.40] | 2.52 [0.46] | 0.03 | 2.70 [0.39] | | 2.63 [0.42] | 0.58 |
| $A\beta_{42}/A\beta_{40}$ (x 100) | 5.17 [0.83] | 6.50 [1.23] | < 0.001 | 5.24 [0.85] | | 6.43 [1.10] | < 0.001 |
| $\ln pTau181$ | 0.50 [0.29] | 0.31 [0.42] | 0.02 | 0.48 [0.32] | | 0.35 [0.39] | 0.29 |

**Supplementary Table 2.** Summary table for participants aged between 65 years and 80 years old used in the baseline and decline models. The Baseline and Decline columns show the characteristics of the same participant measured at baseline, however, for the decline models those with exactly 5 follow-ups from baseline were included, hence the reduced number of participants in the Decline column.

|  | **Baseline** | | | **Decline** | | |
| --- | --- | --- | --- | --- | --- | --- |
|  | **Aβ+** | **Aβ –** | ***P-value*** | **Aβ+** | **Aβ –** | ***P-value*** |
| Number of participants, *n* | 125 | 418 |  | 79 | 289 |  |
| Cognition z-scores, *mean [SD]* | 0.099 [0.83] | 0.22 [0.81] | 0.15 | -2.56 [1.20] | 0.23 [0.88] | < 0.001 |
| Sex, *male* *%* | 52% | 53% | 0.86 | 59% | 54% | 0.41 |
| Age, y, mean [SD] | 73.23 [4.24] | 72.35 [4.13] | 0.04 | 73.54 [4.18] | 72.08 [4.15] | 0.01 |
| Practice effect, mean [SD] | 2.18 [1.61] | 1.84 [1.26] | 0.09 | 2.78 [1.62] | 1.86 [1.30] | 0.10 |
| Education, *mean [SD]* | 14.89 [2.70] | 14.79 [2.52] | 0.62 | 15.57 [2.62] | 14.92 [2.54] | 0.03 |
| ADI, *mean [SD]* | 40.20 [16.23] | 41.23 [18.09] | 0.67 | 35.58 [16.14] | 41.42 [18.35] | 0.23 |
| $\ln GFAP$ | 4.79 [0.41] | 4.54 [0.42] | < 0.001 | 4.73 [0.43] | 4.54 [0.43] | < 0.001 |
| $\ln NfL$ | 3.16 [0.47] | 3.11 [0.46] | 0.21 | 3.17 [0.40] | 3.09 [0.44] | 0.21 |
| $A\beta_{42}/A\beta_{40}$ (x 100) | 5.35 [1.67] | 5.97 [1.22] | < 0.001 | 5.40 [1.15] | 6.00 [1.25] | < 0.001 |
| $\ln pTau181$ | 0.85 [0.46] | 0.53 [0.49] | < 0.001 | 0.82 [0.49] | 0.51 [0.51] | < 0.001 |

**Supplementary Table 3.** Summary table for participants older than 80 years old used in the baseline and decline models. The Baseline and Decline columns show the characteristics of the same participant measured at baseline, however, for the decline models those with exactly 5 follow-ups from baseline were included, hence the reduced number of participants in the Decline column.

|  | ***Baseline*** | | | ***Decline*** | | |
| --- | --- | --- | --- | --- | --- | --- |
|  | **Aβ+** | **Aβ –** | ***P-value*** | **Aβ+** | **Aβ –** | ***P-value*** |
| Number of participants, *n* | 80 | 102 |  | 24 | 41 |  |
| Cognition z-scores, *mean [SD]* | -0.42 [0.89] | -0.30 [0.91] | 0.38 | -0.99 [1.36] | -0.47 [1.13] | 0.16 |
| Sex, *male* *%* | 61% | 58% | 0.64 | 46% | 70% | 0.05 |
| Age, y, mean [SD] | 83.42 [3.09] | 84.27 [3.06] | 0.02 | 82.26 [2.14] | 83.60 [3.12] | 0.07 |
| Practice effect, mean [SD] | 4.66 [2.72] | 4.13 [2.92] | 0.25 | 4.88 [2.36] | 4.12 [2.60] | 0.07 |
| Education, *mean [SD]* | 14.39 [2.88] | 14.21 [3.10] | 0.70 | 13.71 [1.97] | 14.76 [3.48] | 0.18 |
| ADI, *mean [SD]* | 47.17 [16.18] | 46.95 [15.03] | 0.92 | 47.78 [15.85] | 48.79 [13.17] | 0.42 |
| $\ln GFAP$ | 5.04 [0.44] | 4.94 [0.40] | 0.10 | 5.18 [0.43] | 4.89 [0.38] | 0.002 |
| $\ln NfL$ | 3.58 [0.46] | 3.70 [0.44] | 0.12 | 3.59 [0.44] | 3.63 [0.41] | 0.73 |
| $A\beta_{42}/A\beta_{40}$ (x 100) | 5.22 [1.08] | 5.83 [1.49] | 0.01 | 5.07 [1.24] | 5.66 [1.66] | 0.16 |
| $\ln pTau181$ | 0.99 [0.40] | 0.81 [0.43] | 0.004 | 0.94 [0.37] | 0.71 [0.31] | 0.01 |

**Supplementary Table 4.** Ordinary least square linear regression predicting baseline cognitive scores and cognitive decline slopes. The results clearly show an opposite practice effect at baseline for the baseline cognition and the cognitive decline values.

|  | **β** | **std err** | **t** | **P>\|t\|** | **[0.025** | **0.975]** |
| --- | --- | --- | --- | --- | --- | --- |
| **Baseline Cognition Z-scores** | | | | | | |
| Sex | **-0.2220** | 0.042 | -5.241 | 0.000 | -0.305 | -0.139 |
| Age Baseline | **-0.0363** | 0.002 | -17.115 | 0.000 | -0.040 | -0.032 |
| Education | **0.1299** | 0.008 | 15.367 | 0.000 | 0.113 | 0.146 |
| Prior exposure | **0.0689** | 0.014 | 5.068 | 0.000 | 0.042 | 0.096 |
| Amyloid Status | **-0.1786** | 0.057 | -3.141 | 0.002 | -0.290 | -0.067 |
| const | **0.8902** | 0.195 | 4.557 | 0.000 | 0.507 | 1.274 |
| **Cognition Decline (slopes)** | | | | | | |
| Sex | -0.0065 | 0.006 | -1.166 | 0.244 | -0.017 | 0.004 |
| Age Baseline | **-0.0022** | 0.000 | -5.680 | 0.000 | -0.003 | -0.001 |
| Education | 0.0007 | 0.001 | 0.623 | 0.534 | -0.002 | 0.003 |
| Prior exposure | **-0.0146** | 0.002 | -6.780 | 0.000 | -0.019 | -0.010 |
| First time point (years) | -0.0117 | 0.013 | -0.871 | 0.384 | -0.038 | 0.015 |
| Second time point (years) | 0.0094 | 0.016 | 0.602 | 0.548 | -0.021 | 0.040 |
| Third time point (years) | -0.0194 | 0.014 | -1.343 | 0.180 | -0.048 | 0.009 |
| Fourth time point (years) | 0.0170 | 0.011 | 1.524 | 0.128 | -0.005 | 0.039 |
| Fifth time point (years) | -0.0119 | 0.007 | -1.688 | 0.092 | -0.026 | 0.002 |
| Amyloid Status | **-0.0584** | 0.008 | -7.573 | 0.000 | -0.074 | -0.043 |
| const | **0.2239** | 0.040 | 5.579 | 0.000 | 0.145 | 0.303 |

**Supplementary Table 5.** Performance summary of the baseline cognition prediction models with **prior exposures included.** The R2 mean [95% CI]) are shown for the cross-validation test folds. The base models consisted of the feature categories only without socio-demographics.

| **Baseline cognition models** | | | | | |
| --- | --- | --- | --- | --- | --- |
| **Models** | **All** | **Aβ+** | **Aβ–** | **Cohen’s *d*** | ***P*-value** |
| Socio-demographics | 0.38 [0.37 - 0.39] | 0.24 [0.21 - 0.28] | 0.36 [0.34 - 0.37] | 1.28 | < 0.001* |
| **Base models** | | | | | |
| Plasma | 0.17 [0.15 - 0.18] | -0.03 [-0.06 - 0.00] | 0.16 [0.14 - 0.19] | 2.13 | < 0.001* |
| MRI | 0.22 [0.21 - 0.23] | 0.05 [0.02 - 0.09] | 0.19 [0.18 - 0.21] | 1.55 | < 0.001* |
| Cardiovascular | 0.13 [0.12 - 0.14] | 0.03 [-0.00 - 0.06] | 0.11 [0.1 - 0.13] | 1.11 | < 0.001* |
| Lifestyle | 0.03 [0.02 - 0.04] | -0.08 [-0.10 - -0.05] | 0.02 [0.01 - 0.03] | 1.42 | < 0.001* |
| Genetics | 0.01 [-0.0 - 0.02] | -0.07 [-0.10 - -0.04] | -0.01 [-0.02 - -0.01] | 0.76 | 0.01* |
| All inputs | 0.28 [0.27 - 0.29] | 0.11 [0.08 - 0.14] | 0.26 [0.25 - 0.28] | 1.98 | < 0.001* |
| **Base model features + Socio-demographics (Age, sex, education, ADI)** | | | | | |
| Plasma | 0.38 [0.37 - 0.39] | 0.24 [0.21 - 0.27] | 0.37 [0.35 - 0.38] | 1.39 | < 0.001* |
| MRI | 0.40 [0.39 - 0.40] | 0.24 [0.21 - 0.27] | 0.37 [0.36 - 0.39] | 1.70 | < 0.001* |
| Cardiovascular | 0.38 [0.37 - 0.39] | 0.27 [0.24 - 0.30] | 0.36 [0.35 - 0.37] | 1.14 | < 0.001* |
| Lifestyle | 0.38 [0.37 - 0.38] | 0.22 [0.19 - 0.26] | 0.36 [0.35 - 0.38] | 1.49 | < 0.001* |
| Genetics | 0.37 [0.36 - 0.38] | 0.22 [0.18 - 0.25] | 0.34 [0.33 - 0.36] | 1.25 | < 0.001* |
| **Base model features + Socio-demographics + Plasma (Aβ_42_/Aβ_40_, NfL, GFAP, pTau181)** | | | | | |
| Plasma & MRI | 0.40 [0.39 - 0.41] | 0.27 [0.24 - 0.29] | 0.38 [0.36 - 0.4] | 1.53 | < 0.001* |
| Plasma & Cardiovascular | 0.39 [0.38 - 0.40] | 0.27 [0.23 - 0.30] | 0.37 [0.36 - 0.38] | 1.21 | < 0.001* |
| Plasma & Lifestyle | 0.38 [0.37 - 0.39] | 0.23 [0.20 - 0.27] | 0.37 [0.36 - 0.39] | 1.51 | < 0.001* |
| Plasma & Genetics | 0.37 [0.36 - 0.38] | 0.27 [0.24 - 0.30] | 0.36 [0.35 - 0.37] | 1.10 | < 0.001* |
| All inputs | 0.40 [0.39 - 0.41] | 0.29 [0.26 - 0.31] | 0.38 [0.37 - 0.39] | 1.38 | < 0.001* |

GFAP = Glial fibrillary acidic protein, NfL = Neurofilament light, pTau181 = Phosphorylated tau, ADI = Area Deprivation Index National Rank.

**P-value* < 0.05

**Supplementary Table 6.** Performance summary of the cognitive decline (slope) prediction models with **prior exposures included.** The R2 mean [95% CI]) are shown for the cross-validation test folds. The base models consisted of the feature categories only without socio-demographics.

| **Cognitive decline (slope) models** | | | | | |
| --- | --- | --- | --- | --- | --- |
| **Models** | **All** | **Aβ+** | **Aβ–** | **Cohen’s *d*** | ***P*-value** |
| Socio-demographics | 0.24 [0.22 - 0.25] | -0.02 [-0.09 - 0.04] | 0.20 [0.18 - 0.22] | 1.31 | 0.004* |
| **Base models** | | | | | |
| Plasma | 0.22 [0.2 - 0.24] | 0.05 [-0.0 - 0.10] | 0.13 [0.11 - 0.15] | 0.59 | 0.004* |
| MRI | 0.24 [0.22 - 0.26] | 0.12 [0.07 - 0.17] | 0.20 [0.18 - 0.21] | 0.56 | 0.06 |
| Cardiovascular | 0.21 [0.19 - 0.23] | 0.02 [-0.03 - 0.07] | 0.16 [0.14 - 0.18] | 1.04 | < 0.001* |
| Lifestyle | 0.16 [0.14 - 0.18] | 0.11 [0.06 - 0.17] | 0.11 [0.09 - 0.13] | 0.00 | 0.59 |
| Genetics | 0.16 [0.14 - 0.18] | -0.07 [-0.13 - -0.0] | 0.11 [0.09 - 0.14] | 1.06 | < 0.001* |
| All inputs | 0.29 [0.27 - 0.30] | 0.12 [0.07 - 0.18] | 0.20 [0.18 - 0.21] | 0.54 | 0.01* |
| **Base model features + Socio-demographics (Age, sex, education, ADI)** | | | | | |
| Plasma | 0.23 [0.22 - 0.25] | 0.04 [-0.02 - 0.1] | 0.18 [0.16 - 0.2] | 0.88 | < 0.001* |
| MRI | 0.23 [0.22 - 0.24] | 0.13 [0.08 - 0.17] | 0.22 [0.2 - 0.24] | 0.75 | < 0.001* |
| Cardiovascular | 0.24 [0.22 - 0.25] | 0.04 [-0.01 - 0.09] | 0.19 [0.18 - 0.21] | 1.13 | < 0.001* |
| Lifestyle | 0.19 [0.18 - 0.21] | 0.1 [0.05 - 0.14] | 0.19 [0.17 - 0.21] | 0.80 | 0.001* |
| Genetics | 0.20 [0.18 - 0.22] | 0.03 [-0.03 - 0.09] | 0.18 [0.16 - 0.21] | 1.01 | < 0.001* |
| **Base model features + Socio-demographics + Plasma (Aβ42 / Aβ40, NfL, GFAP, pTau181)** | | | | | |
| Plasma & MRI | 0.26 [0.25 - 0.28] | 0.18 [0.13 - 0.23] | 0.22 [0.21 - 0.24] | 0.29 | 0.15 |
| Plasma & Cardiovascular | 0.27 [0.25 - 0.29] | 0.09 [0.01 - 0.17] | 0.19 [0.17 - 0.21] | 0.53 | 0.02 |
| Plasma & Lifestyle | 0.24 [0.22 - 0.25] | 0.07 [0.02 - 0.12] | 0.19 [0.18 - 0.21] | 0.90 | < 0.001* |
| Plasma & Genetics | 0.24 [0.22 - 0.26] | 0.04 [-0.02 - 0.1] | 0.18 [0.16 - 0.2] | 0.89 | < 0.001* |
| All inputs | 0.27 [0.25 - 0.28] | 0.16 [0.12 - 0.21] | 0.21 [0.2 - 0.23] | 0.39 | 0.05 |

GFAP = Glial fibrillary acidic protein, NfL = Neurofilament light, pTau181 = Phosphorylated tau, ADI = Area Deprivation Index National Rank.

**P-value*  < 0.05

- End of document
